# Five years of advances in electrochemical analysis of protein biomarkers in lung cancer: a systematic review

**DOI:** 10.3389/fchem.2024.1390050

**Published:** 2024-05-03

**Authors:** Matías Regiart, Martín A. Fernández-Baldo, Bernardino Alcázar Navarrete, Concepción Morales García, Beatriz Gómez, Gonzalo R. Tortella, Teresa Valero, Francisco Gabriel Ortega

**Affiliations:** ^1^ Instituto de Química San Luis (INQUISAL), Departamento de Química, Universidad Nacional de San Luis, CONICET, San Luis, Argentina; ^2^ IBS Granada, Institute of Biomedical Research, Granada, Spain; ^3^ Pulmonology Unit, Hospital Universitario Virgen de las Nieves, Granada, Spain; ^4^ CIBERES, Instituto de Salud Carlos III, Madrid, Spain; ^5^ Centro de Excelencia en Investigación Biotecnológica Aplicada al Medio Ambiente (CIBAMA), Facultad de Ingeniería y Ciencias, Universidad de La Frontera, Temuco, Chile; ^6^ Departamento de Ingeniería Química, Facultad de Ingeniería y Ciencias, Universidad de La Frontera, Temuco, Chile; ^7^ Department of Medicinal and Organic Chemistry and Excellence Research Unit of “Chemistry Applied to Biomedicine and the Environment”, Faculty of Pharmacy, University of Granada, Granada, Spain; ^8^ GENYO, Centre for Genomics and Oncological Research, Pfizer/University of Granada/Andalusian Regional Government, Granada, Spain; ^9^ UGC Cartuja, Distrito Sanitario Granada Metropolitano, Granada, Spain

**Keywords:** electrochemical methods, biosensors, lung cancer, diagnosis, biomarkers, clinical analysis

## Abstract

Lung cancer is the leading cause of cancer death in both men and women. It represents a public health problem that must be addressed through the early detection of specific biomarkers and effective treatment. To address this critical issue, it is imperative to implement effective methodologies for specific biomarker detection of lung cancer in real clinical samples. Electrochemical methods, including microfluidic devices and biosensors, can obtain robust results that reduce time, cost, and assay complexity. This comprehensive review will explore specific studies, methodologies, and detection limits and contribute to the depth of the discussion, making it a valuable resource for researchers and clinicians interested in lung cancer diagnosis.

## 1 Introduction

Lung cancer is the leading cause of cancer death in both men and women and the second most common cancer after female breast cancer (Li et al., 2023). Importantly, in the United States, the number of lung cancer cases and lung cancer-related deaths is decreasing due to reduced smoking in the population and the advances in early detection and treatments (Schabath and Cote, 2019). As lung cancer is mostly diagnosed in people older than 65 years, the American Cancer Society recommends early lung cancer screening testing in people after 50 years old with a smoking history in the last 15 years (Ravi et al., 2022). A yearly low-dose computed tomography (CT) scan to screen for lung cancer is recommended (Tanoue et al., 2015), but the high cost of this technique limits lung cancer screening. The lack of alternative cost-effective, sensitive, and specific screening methods for the early detection of lung cancer points out the urgent need for minimally invasive methods for early biomarker detection. Such a tool would provide a diagnostic or a complementary diagnostic tool for lung cancer in the at-risk population (Seijo et al., 2019).

In recent years, several protein biomarkers have been investigated as complementary to low-dose CT or as a direct diagnosis of lung cancer (Triphuridet et al., 2018). In addition to cancer diagnosis, circulating protein biomarkers are useful for monitoring and predicting treatment response, an application that has been validated for most currently identified biomarkers (Zhang J. et al., 2021). Most of these biomarkers are low-abundance proteins, requiring high sensibility and specificity, sophisticated equipment, and specialized personnel (Huang et al., 2016). To overcome these analytical limitations for lung cancer screening, electrochemical analysis emerges as a revolutionary technology to obtain robust results, reducing the time, cost, and assay complexity (Zhu et al., 2015). Electrochemical biosensors are devices containing impedimetric, amperometric, potentiometric, conductometric, or voltammetric transducers for the analysis of biological biomarkers (Yadav et al., 2022). A conventional three-electrode electrochemical cell contains the working electrode (WE), the counter electrode (CE), and the reference electrode (RE) (Brett and Brett, 1993). It is fabricated from stable materials, and the chemical composition of the electrodes significantly affects the analytical parameters during measurement. Current trends in analytical chemistry include the modification of the electrode materials and the functionalization of electrode surfaces as a means to improve analysis time, costs, portability, limits of detection, and quantification (Tang and Ma, 2017).

In this systematic review, we have identified advances in the analysis of lung cancer protein biomarkers by electrochemical methods during the last 5 years, focusing on three classifications of lung cancer (total lung cancer, non-small cell lung cancer, and small cell lung cancer), as well as the type of electrochemical transducer, the electrode modifications, and analytical parameters.

## 2 Methods

The preferred reporting items for systematic reviews and meta-analysis (PRISMA) guidelines were followed to perform this systematic review (Page et al., 2021). We established our literature search from 1 January 2017 to 30 July 2022 using the SCOPUS, PubMed, ScienceDirect, and Web of Science databases. The search terms were combinations of the following expression in the title, abstract, or keywords: lung AND (disease OR cancer) AND electrochemical NOT review.

Inclusion criteria: Original research publications with an abstract that presented experimental results that applied electrochemical detection for lung cancer diagnosis. Studies report qualitative and quantitative data and refer to the analyzed biomarker, kind of electrochemical transducer, composition, and modification of electrodes. Exclusion criteria: Original research reported in a language other than English, articles without electrochemical analysis, non-protein biomarkers, non-cancer disease, infectious disease, or cell culture studies.

## 3 Results

### 3.1 Results of PRISMA statement evidence search and selection

**FIGURE 1:**
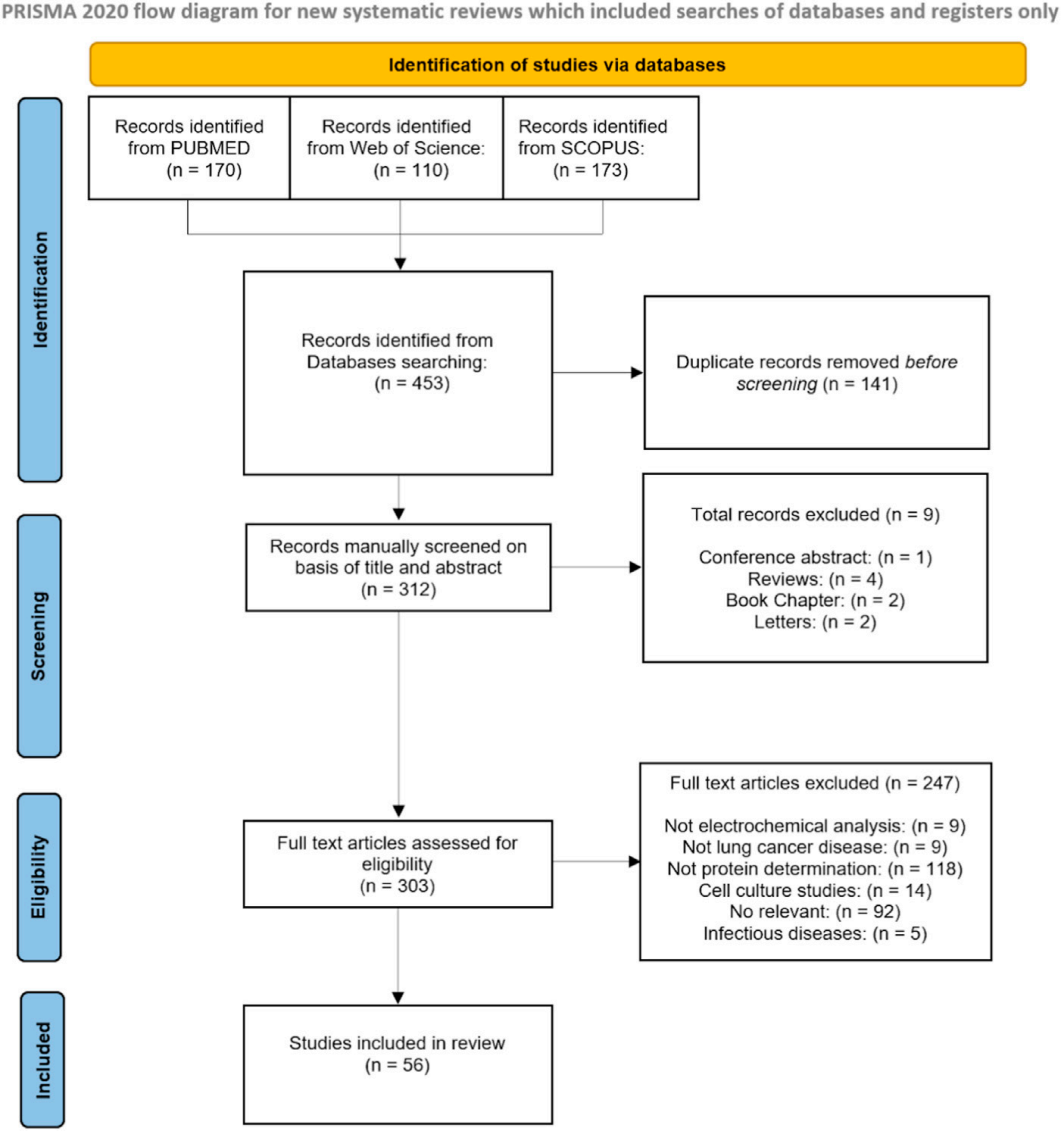
PRISMA flow diagram.

The PUBMED search identified 170 publications; another 110 were found in the Web of Science, and 173 were detected using the SCOPUS databases. The total number of identified records was 453, of which 141 were duplicates that were removed before screening. The remaining 312 records were manually evaluated based on title and abstract. Nine records were excluded as they were conference abstracts (*n* = 1), reviews (*n* = 4), book chapters (*n* = 2), or letters (*n* = 2). Finally, 303 articles were assessed for eligibility, of which 246 did not meet the inclusion or exclusion criteria as they reported non-electrochemical analysis (*n* = 9), not related to lung cancer disease (*n* = 9), no protein determination (*n* = 117), were cell culture studies (*n* = 14), were related to infectious diseases: (*n* = 5), or were not relevant (*n* = 85). A detailed diagram of the selection process can be seen below in Figure 1.

**FIGURE 2:**
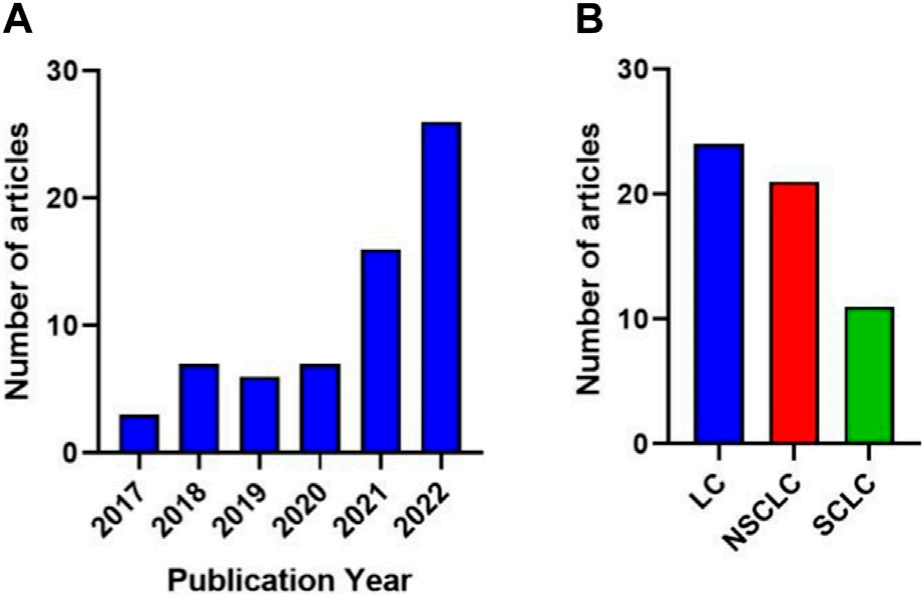
**(A)** The number of publications exponentially increased from 2020 until now. **(B)** Number of publications according to the type of tumor: total lung cancer (LC), non-small cell lung cancer (NSCLC), and small cell lung cancer (SCLC).

From the final 57 studies included in this review, the number of publications exponentially increased from 2020 until now (Figure 2A). The studies were classified into three categories according to the type of tumor: total lung cancer (LC), which comprises indistinctly all kinds of LC, non-small cell lung cancer (NSCLC), and small cell lung cancer (SCLC) (Figure 2B). Table 1 shows a list of abbreviations of methods and biomarkers.

**TABLE 1:** List of abbreviations of methods and biomarkers.

Method		Biomarker	
CA	Chronoamperometry	AFP	Alpha-fetoprotein
CV	Cyclic voltammetry	AGR2	Anterior gradient 2
DPV	Differential pulse voltammetry	CA125	Cancer antigen 125
ECL	Electrogenerated chemiluminescence	CEA	Carcinoembryonic antigen
EIS	Electrochemical impedance spectroscopy	EGFR	Epidermal growth factor receptor
NIE	Nanoparticle impact electrochemistry	GM2AP	Ganglioside GM2 activator protein
PEC	Photoelectrochemical cell	ProGRP	Pro-gastrin-releasing peptide
SECM	Scanning electrochemical microscopy	GzmB	Granzyme B
SWV	Square wave voltammetry	NAP2	Neutrophil-activating protein-2
EDL	Electric double layer	NSE	Neuron-specific enolase
PEC	Photoelectrochemical	MUC1	Mucin 1
ASV	Anodic stripping voltammetry	PDGFR	Platelet-derived growth factor receptor
		PDL1	Programmed death-ligand 1
		Sam68	Src associated in mitosis 68-kDa protein
		SFTPB	Pulmonary-surfactant protein
		SOX2	Transcription factor SOX-2
		uPAR	Urokinase-type plasminogen activator receptor
		EpCAM	Anti-epithelial cell adhesion molecule
		p53	Protein53
		CD44	Cell-surface glycoprotein
		CD63	Protein associated with membranes of intracellular vesicles
		CD9	Protein member of the transmembrane 4 superfamily
		CD81	Cluster of differentiation 81
		CYFRA21.1	Cytokeratin fragment

## 4 Electrochemical detection methodologies for different lung cancer types

### 4.1 Total lung cancer (LC)

**FIGURE 3:**
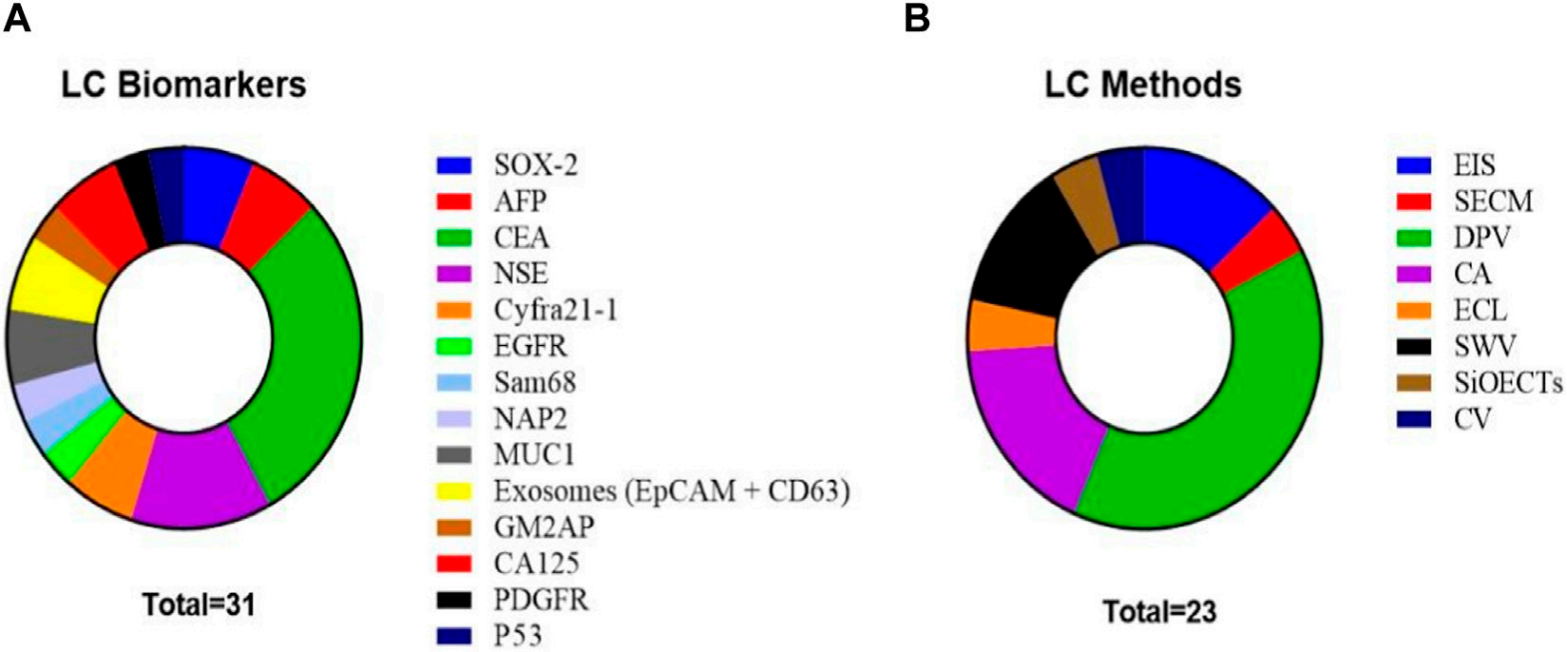
**(A)** Total LC biomarkers studied in the reviewed articles. **(B)** Electrochemical methods used in the reviewed articles for biomarker analysis in all forms of lung cancer.

In the last 5 years, a wide range of lung cancer biomarkers, such as sex-determining region Y-box 2 (SOX-2), alpha-fetoprotein (AFP), carcinoembryonic antigen (CEA), neuron-specific enolase (NSE), cytokeratin-19-fragment (CYFRA21-1), CD44, cell-surface glycoprotein (CD44), epidermal growth factor receptor (EGFR), Src-associated protein in mitosis 68 (Sam68), neutrophil-activating peptide 2 (NAP2), LC-18, mucin 1 (MUC1), ganglioside GM2 activator protein (GM2AP), cancer antigen 125 (CA125), platelet-derived growth factor receptor (PDGFR), exosomes (epithelial cell adhesion molecule (EpCAM) + CD63), and P53, have been studied by electrochemical detection methodologies (Figure 3A). Our systematic search found 24 studies reporting various electrochemical techniques (Figure 3B), some of which use modifications and functionalization of electrodes in order to improve the analytical properties for real clinical samples (serum, plasma, sputum, cells, or urine) obtained from patients with lung cancer.

#### 4.1.1 SOX-2 and CEA

Sex-determining region Y-box 2 (SOX-2) has been considered a biomarker of lung cancer. SOX-2 is a transcription factor key to maintaining stem cells, essential for early development, that is expressed in adult lung tissue (Lu et al., 2010). SOX-2 overexpression has been found in all types of lung cancer (SCLC and NSCLC). While a cut-off for its clinical application has not been standardized, it is a good biomarker candidate and also a potential therapeutic target (Karachaliou et al., 2013). Aydin and Sezgintürk (2017) reported the electrochemical detection of SOX-2 in serum samples with a novel immunosensor via the modification of a disposable indium tin oxide (ITO) thin film-based electrode with carboxyethylsilanetriol (CTES). Later, Regiart et al. (2020) proposed a microfluidic electrochemical immunosensor for SOX-2, specifically, a modified electrode with gold nanoporous structures (NPAu) that uses the dynamic hydrogen bubble template (DHBT) method.

Carcinoembryonic antigen (CEA) is a glycoprotein that is normally produced in very low levels by some cells in the body, but its levels can rise in the blood of individuals with certain types of cancer, including lung cancer, where it has a standardized cut-off of 3.2 ng/mL (Okamura et al., 2013). CEA is one of the most used electrochemical biomarkers, although its use as a single biomarker for disease diagnosis and prognosis is still controversial (Grunnet and Sorensen, 2012). As a first example, Paimard et al., 2020 presented an electrochemical immunosensor based on carbon paste electrodes (CPE) modified with honey nanofibers (HNF) by electrospinning, which in turn were decorated with gold nanoparticles (GNPs) by electrodeposition and multi-wall carbon nanotubes (MWCNTs) functionalized with carboxylic acid groups. Then, Zhou et al. (2022) described an electrochemical biosensor based on dual-signal amplification through electrically mediated atom transfer radical polymerization (eATRP) to significantly improve the level of detection (LOD). The subsequent covalent conjugation of 2-bromo-2-methylpropionic acid (BMP) allowed eATRP initiation to graft numerous ferrocene methyl methacrylate (FMMA) monomers onto the modified electrode under electrocatalysis. A rather interesting design by Wang X. et al. (2022) describes a “dual-signal off” electrochemical immunosensor based on the combination of two functionalized solid supports. On one hand, Prussian blue nanoparticles (PBNPs) covered with gold nanoparticles-reduced graphene oxide (Au-rGO), and, on the other hand, mesoporous silica nanoshells coated with copper peroxide (CuO_2_@SiO_2_) nanocomposites allow the release of Cu^2+^ and H_2_O_2_ under acidic conditions. The generated Cu^2+^ replaces the high-spin iron (Fe^III^) in the PBNPs, which in turn reduces the PBNPs’ oxidation peak current.

#### 4.1.2 CEA combinations

**FIGURE 4:**
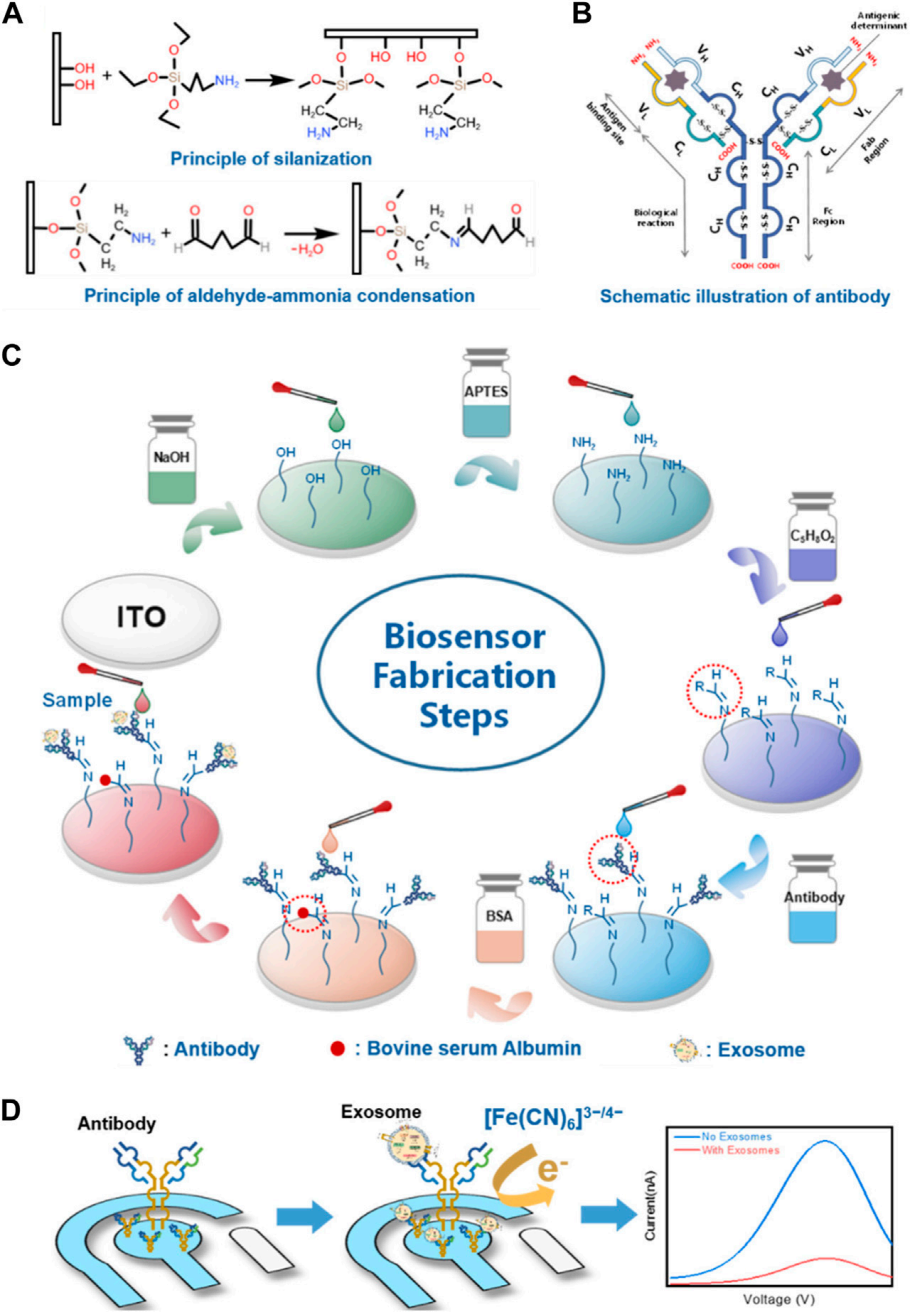
Schematic representation of the designed immunosensor for exosome biomarker detection. **(A)** Principle of the silanization process by APTES and aldehyde–ammonia condensation by glutaraldehyde on the hydroxylated working electrode. **(B)** Schematic illustration of antibody. **(C)** Fabrication procedures of the electrochemical immunosensor. **(D)** Principle of differential pulse voltammetry for the detection of exosomes. Reprinted with permission from Fan et al. (2022) (https://creativecommons.org/licenses/by/4.0/).

Although an accurate determination of CEA may be used as a biomarker in a clinical setup, the accuracy of cancer diagnosis can be increased by detecting several biomarkers in a given sample. Our search rendered several assays developed for multiple and simultaneous biomarker determination in addition to CEA. Ning et al. (2018) developed an immune sandwich structure with antibodies for CEA, alpha-fetoprotein (AFP), neuron-specific enolase (NSE), and cytokeratin-19-fragment (CYFRA21-1) on a microarray coupled with scanning electrochemical microscopy (SECM). Upon addition of sample proteins and HRP-labeled antibodies, HRP catalyzed the oxidation of hydroquinone (H_2_Q) to form benzoquinone (BQ) in the presence of H_2_O_2_ on the spots. To avoid the need for cleanroom fabrication, Wang et al. (2019) developed a paper-based electrochemical aptasensor for the detection of carcinoembryonic antigen (CEA) and NSE. To achieve the simultaneous detection of the two markers, the authors used two microfluidic channels and two screen-printed carbon ink working electrodes. The electrodes were modified with amino-functional graphene-thionin-gold nanoparticles (NG-THI-AuNPs) and Prussian blue-poly(3,4-ethylenedioxythiophene)-AuNPs (PB-PEDOT-AuNPs) nanocomposites. A different approach to detecting two biomarkers was used by Li et al. (2020), who proposed a novel electrical signal difference strategy for CEA and AFP detection employing a glassy carbon electrode (GCE). Recently, Zhang R. et al. (2022) broadened the type of sample that could be analyzed by developing a portable multi-channel interdigitated organic electrochemical transistor (SiOECTs) device for noninvasive CEA, NSE, and cancer antigen 125 (CA125) in sputum. Later, simultaneous electrochemical immunodetection of CEA and CA125 was reported by Liu et al. (2022), who developed an immunosensor based on copper-tetrakis (4-carboxyphenyl) porphyrin (Cu-TCPP) nanomaterials and toluidine blue (TB) plus PB as the indicators with a different redox signal. As a last example, Fan et al. (2022) approached the multiplexing detection using a tri-channel electrochemical immunobiosensor for CEA, NSE, and CYFRA21-1 detection in exosomes by using indium tin oxide electrode (ITO) functionalized with (3-aminopropyl) triethoxysilane (APTES) (Figure 4).

#### 4.1.3 Surface glycoproteins

Several cell-surface glycoproteins that are overexpressed in lung cancer have been used as biomarkers in cancer diagnosis, and their applicability in serum analysis needs better understanding.

For CD44, a transmembrane glycoprotein that is widely expressed on the surface of cells in the majority of normal and carcinomatous tissues, Białobrzeska et al. (2021) used hyaluronic acid (HA) as a sensing element to design a label-free electrochemical sensor for CD44 based on ligand–protein interactions. A representative approach in the liquid biopsy field is the work by Zhang J. et al. (2021), who reported a micropatterned electrochemical aptasensor integrated with a hybridization chain reaction (HCR) signal amplification method using anti-CD63 aptamer-functionalized gold microelectrodes for EVs capture and anti-epithelial cell adhesion molecule (EpCAM) aptamers for detection. CD63 is a member of the tetraspanin family and a generalized biomarker of total EVs. For its part, EpCAM is a molecular adhesion protein overexpressed in all epithelial-origin cancers, including lung cancer; its combined analysis results in an assessment of circulating epithelial EVs. You et al. (2022) constructed an electrochemical biosensor based on Ti_2_CT_x_ MXene nanosheets decorated with a gold nanoarray for exosome detection using anti-EpCAM aptamers as sensing elements.

Another protein employed as a biomarker is mucin 1 (MUC1), a glycoprotein member of the mucin family that has been found to be increased in lung cancer; its anti-adhesion characteristics and cell signaling implications support the role of MUC1 in the development of human lung cancer and its potential as a biomarker (Saltos et al., 2018). The electrochemical detection of MUC1 was reported by Zhao et al. (2021), who developed a novel hybrid peroxidase mimetic structure to amplify the sensitivity towards the decomposition of H_2_O_2_ by anchoring Cu_7_S_4_ and AuNPs on a porphyrin-based porous organic polymer (PPOP) and catalytic hairpin assembly (CHA) to amplify signals. Xie et al. (2022) proposed an electrochemical ratiometric aptasensor with intrinsic self-calibration properties. Co-metal-organic frameworks (MOFs) were employed as signal substances. The gold nanoparticles@black phosphorus (BP) comprising the electrode substrate was modified via an Au-S bond with DNA nano-tetrahedrons (DTN) containing MUC1 aptamers.

#### 4.1.4 Membrane receptors

**FIGURE 5:**
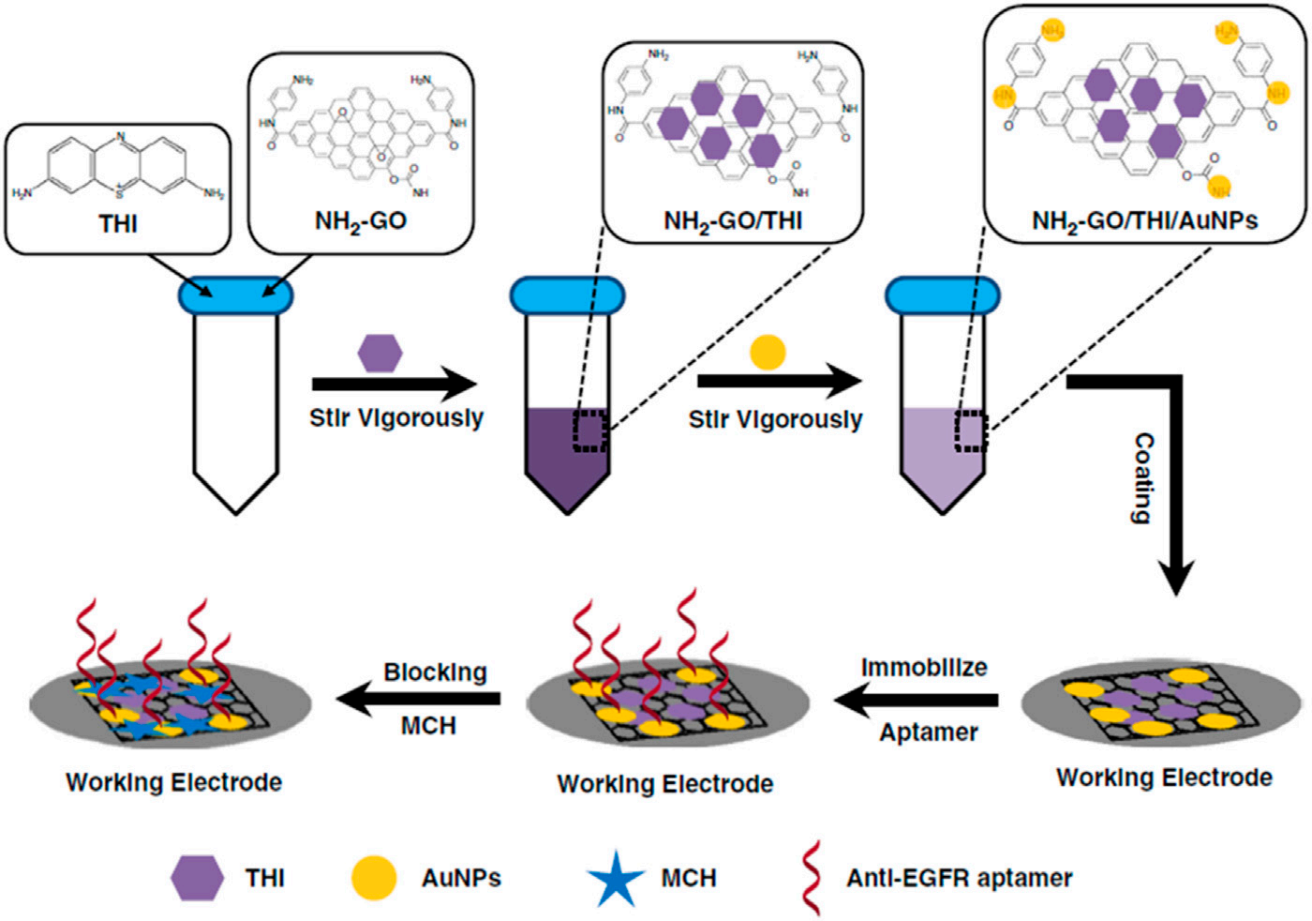
Modification process of the origami-paper-based EGFR aptasensor. Reprinted with permission from Wang et al. (2020) (https://creativecommons.org/licenses/by/4.0/).

Overexpressed growth factor receptors are associated with cancer development and have been studied as biomarkers. Wang et al. (2020) designed an electrochemical origami paper-based aptasensor for label-free detection of epidermal growth factor receptor (EGFR) by employing anti-EGFR aptamers as the bio-recognition element. The hydrophobic areas were created by wax printing, and the electrodes were screen printed. Amino-functionalized graphene/thinine/gold nanoparticle nanocomposites were used to modify the carbon ink working electrode and accommodate the anti-EGFR aptamers through Au-S bonds (Figure 5). Rejeeth et al. (2022) used poly (acrylic acid) (PAA) as an affinity bait molecule to capture and detect the platelet-derived growth factor receptor (PDGFR).

#### 4.1.5 Others

Sumithra et al. (2020) fabricated an impedimetric biosensor for the splicing factor called Src-associated protein in mitosis 68 (Sam68) detection. Sam68 is overexpressed as lung cancer advances to metastatic stages and thus can be considered a biomarker of disease progression. In this case, the affinity capture molecule is an anti-Sam68 antibody, which is immobilized via glutaraldehyde over a GCE modified with polyaniline (PANI). Chen et al. (2020) detected the early LC biomarker neutrophil-activating peptide 2 (NAP2) with two variants of a signal-off/on electrochemiluminescence (ECL) deoxyribosensors. Another example of immunoaffinity capture and detection is the electrochemical immunosensor designed by Kuntamung et al. (2021) for GM2 activator protein (GM2AP) detection. GM2AP is a novel urinary biomarker in LC patients, who can show over a five-fold increase in this biomarker compared to healthy controls. In brief, screen-printed carbon electrodes were modified with polyethyleneimine-coated gold nanoparticles (PEI-AuNP), phosphomolybdic acid (PMA), and anti-GM2AP antibodies. GM2AP detection is based on a decrease in the differential pulse voltammetry (DPV) current response of PMA redox probes proportional to the GM2AP concentrations. Finally, following the immunoaffinity capture strategy, Deepa et al. (2022) reported an amperometric immunosensor to detect p53, a tumor suppressor gene known as the “guardian of the genome” because its principal function is the control of repairing DNA that is highly activated in cancer. This rather simple device was fabricated by immobilizing anti-p53 antibodies onto a pencil graphite electrode (PGE) via adsorption. Table 2 summarizes the principal characteristics of reviewed articles in total LC.

**TABLE 2:** Summarize the principal characteristics of reviewed articles in total LC.

Reference	Biomarker	Redox signaling species	Method	Modification	Recognition	LOD	Linear range	Sample
Aydın and Sezgintürk (2017)	SOX-2	[Fe(CN)_6_]^3−/4−^	EIS	CTES/ITO	Antibodies	7 fg/mL	25 fg/mL–2 pg/mL	Serum
Ning et al. (2018)	CEA	PBQ	SECM	Pt ME	Antibodies	40 ng/mL	5 ng/mL–1 μg/mL	—
	AFP					42 ng/mL		
	NSE					69 ng/mL		
	CYFRA21-1					67 ng/mL		
Zhang et al. (2019)	CD44	[Fe(CN)_6_]^3−/4−^	DPV	MWCNTs-PDDA-HA/ITO	HA	5.94 pg/mL	0.01–100 ng/mL	serum
Wang et al. (2019)	CEA	—	DPV	NG-THI/AuNPs/CE	Aptamer	2 pg/mL	0.01–500 ng/mL	Serum
	NSE			PB-PEDOT/AuNPs/CE		10 pg/mL	0.05–500 ng/mL	
Li et al. (2020)	CEA	[Fe(CN)_6_]^3−/4−^	CA	PdAg MNS/GCE	Antibodies	0.5 pg/mL	0.001–40 ng/mL	Serum
	AFP					1 pg/mL	0.005–100 ng/mL	
Paimard et al. (2020)	CEA	[Fe(CN)_6_]^3−/4−^	EIS	MWCNTs/GNPs/HNF/CPE	Antibodies	0.09 ng/mL	0.4–125 ng/mL	Serum
Regiart et al. (2020)	SOX-2	CT	CA	NPAu/Au	Antibodies	30 pg/mL	0.11–30 ng/mL	Serum
Wang et al. (2020)	EGFR	—	DPV	NH_2_-GO/THI/AuNP/CE	Aptamer	5 pg/mL	0.05–200 ng/mL	Serum
Sumithra et al. (2020)	Sam68	[Fe(CN)_6_]^3−/4−^	EIS	PANI/Glu/Sam68-Ab/BSA/GCE	Antibodies	10.5 pg/mL	1–5 μg/mL	Cell extract
Chen et al. (2020)	NAP2	—	ECL	5′-NH_2_-(CH_2_)_6_-NBAT-Ru/GCE	Aptamer	0.008 pM	0.01–0.1 pM	Plasma
				5′-SH-(CH_2_)_6_-NBAT-Ru/GE		0.001 pM	0.001–0.1 pM	
Zhao et al. (2021)	MUC1	TMB	DPV	AuNPs@Cu_7_S_4_@Cu/Mn-AzoPPOP	Aptamer	0.72 fg/mL	1 fg/mL to 10 pg/mL	Serum
			CA			0.82 fg/mL		
Zhang et al. (2021b)	Exosomes (EpCAM + CD63)	TMB	CA	Ti/Au	Aptamer	5×10^2^ ex/mL	2.5 × 10^3^–1 × 10^7^ ex/mL	Serum
Kuntamung et al. (2021)	GM2AP	[Fe(CN)_6_]^3−/4−^	DPV	PMA/PEI-AuNP/SPCE	Antibodies	0.51 pg/mL	0.005–25 ng/mL	Urine
							25–400 ng/mL	Serum
Zhou et al. (2022)	CEA	Cu^II^Br/Me_6_TREN^+^	SWV	FMMA/BMP/Apt2-PEI/CEA/MCH/Apt1/Au	Aptamer	70.17 fg/mL	10^-3^–10^2^ ng/mL	Serum
Zhang et al. (2022a)	CEA	—	EDL	AgNWs@PEDOT:PSS Cr/Au	Aptamer	1 pg/mL	1 pg/mL–1 μg/mL	Sputum
	NSE					0.1 pg/mL	0.1 pg/mL–1 μg/mL	
	CA125					50 μU/mL	50 μU/mL–500 U/mL	
Wang et al. (2022b)	CEA	PBNPs	SWV	PBNPs/Au-rGO/GCE	Antibodies	0.0032 pg/mL	0.01 pg/mL–80 ng/mL	Serum
Liu et al. (2022)	CEA	[Fe(CN)_6_]^3−/4−^	SWV	MWCNT/GCE	Antibodies	0.03 ng/mL	0.1–160 ng/mL	Serum
	CA125					0.05 U/mL	0.5–200 U/mL	
Rejeeth et al. (2022)	PDGFR	[Fe(CN)_6_]^3−/4−^	DPV	MWCNTs−PDDA−PAA/ITO	PAA	1.5 pg/mL	1-10,000 ng/mL	Serum
You et al. (2022)	Exosomes (EpCAM + CD63)	[Fe(CN)_6_]^3−/4−^	DPV	Au–Ti_2_CTx MXene	Aptamer	58 ex/μL	1 × 10^2^–1 × 10^7^ ex/μL	Serum
Deepa et al. (2022)	P53	Zobell’s solution	CV	PGE	Antibodies	10 pg/mL	10 pg/mL–10 ng/mL	Serum
Fan et al. (2022)	CEA	[Fe(CN)_6_]^3−/4−^	DPV	ITO	Antibodies	10^−4^ ng/mL	10^-3^–10 ng/mL	Cell lines
	NSE						10^-4^–10^2^ ng/mL	
	CYFRA21-1						10^-3^–10^2^ ng/mL	
Xie et al. (2022)	MUC 1	[Fe(CN)_6_]^3−/4−^	DPV	AuNPs@BP/GCE	Aptamer	1.34 fM	0.004–400 pM	Serum

CA, chronoamperometry; CV, cyclic voltammetry; DPV, differential pulse voltammetry; ECL, electrogenerated chemiluminescence; EIS, electrochemical impedance spectroscopy; SECM, scanning electrochemical microscopy; SWV, square wave voltammetry; EDL, electric double layer; AFP, alpha-fetoprotein; CA125, cancer antigen 125; CEA, carcinoembryonic antigen; EGFR, epidermal growth factor receptor; GM2AP, ganglioside GM2 activator protein; NAP2, neutrophil-activating protein-2; NSE, neuron-specific enolase; CYFRA21.1, cytokeratin fragment; MUC1, mucin 1; EpCAM, anti-epithelial cell adhesion molecule; PDGFR, platelet-derived growth factor receptor; Sam68, Src-associated in mitosis 68 kDa protein; SOX2, transcription factor SOX-2; p53, Protein53; CD44, cell-surface glycoprotein; CD63, protein associated with membranes of intracellular vesicles; PBQ, p-benzoquinone; CT, catechol; TMB, 3,3′,5,5′- tetramethylbenzidine; Cu^II^Br/Me_6_TREN^+^, Cu^II^Br/Tris (2-dimethylaminoethyl) amine; PBNPs, Prussian blue nanoparticles; CTES/ITO, carboxyethylsilanetriol/indium tin oxide; Pt ME, Pt microelectrode; MWCNTs-PDDA-HA/ITO, multi-wall carbon nanotubes- poly(diallyldimethylammonium chloride)-hyaluronic acid/indium tin oxide; NG-THI/AuNPs/CE, amino functional graphene-thionin-gold nanoparticles/carbon electrode; PB-PEDOT/AuNPs/CE, Prussian blue-poly (3,4-ethylenedioxythiophene)-AuNPs nanocomposites/carbon electrode; PdAg MNS/GCE, PdAg mesoporous nanosphere/glassy carbon electrode; MWCNTs/GNPs/HNF/CPE, multi-wall carbon nanotubes/gold nanoparticles/honey nanofibers/carbon paste electrode; NPAu/Au, gold nanoporous/Au; NH_2_-GO/THI/AuNP/CE, amino-functionalized graphene/thionine/gold particle nanocomposites; PANI/Glu/Sam68-Ab/BSA/GCE, polyaniline/glutaraldehyde/Sam68-antibody/bovine serum albumin/glassy carbon electrode; 5′-NH_2_-(CH_2_)_6_-NBAT-Ru/GCE, 5′-NH_2_-(CH_2_)_6_-aptamer-Ru/glassy carbon electrode; 5′-SH-(CH_2_)_6_-NBAT-Ru/GE, 5′-SH-(CH_2_)_6_-aptamer-Ru/glassy electrode; AuNPs@Cu_7_S_4_@Cu/Mn-AzoPPOP, AuNPs@Cu_7_S_4_@Cu/Mn-azoporphyrin-based porous organic polymers; PMA/PEI-AuNP/SPCE, phosphomolybdic acid/polyethyleneimine-coated gold nanoparticle/screen-printed carbon electrode; FMMA/BMP/Apt2-PEI/CEA/MCH/Apt1/Au, ferrocene methyl methacrylate/2-bromo-2-methylpropionic acid/aptamer 2-polyethyleneimine/CEA/6-mercapto-1-hexanol/aptamer1/Au; AgNWs@PEDOT, PSS Cr/Au, silver nanowires@poly(3,4-ethylenedioxythiophene):poly(styrenesulfonate) Cr/Au; PBNPs/Au-rGO/GCE, Prussian blue nanoparticles/gold nanoparticles-reduced graphene oxide/glassy carbon electrode; MWCNT/GCE, multi wall carbon nanotubes/glassy carbon electrode; MWCNTs-PDDA-PAA/ITO, multi-wall carbon nanotubes-poly(diallyldimethylammonium chloride)-poly(acrylic acid)/indium tin oxide; Au-Ti_2_CTx MXene, Au-Ti_2_CTx two-dimensional MXene membranes; PGE, pencil graphite electrode; ITO, indium tin oxide; AuNPs@BP/GCE, gold nanoparticles@black phosphorus/glassy carbon electrode.

### 4.2 Non-small cell lung cancer (NSCLC)

**FIGURE 6:**
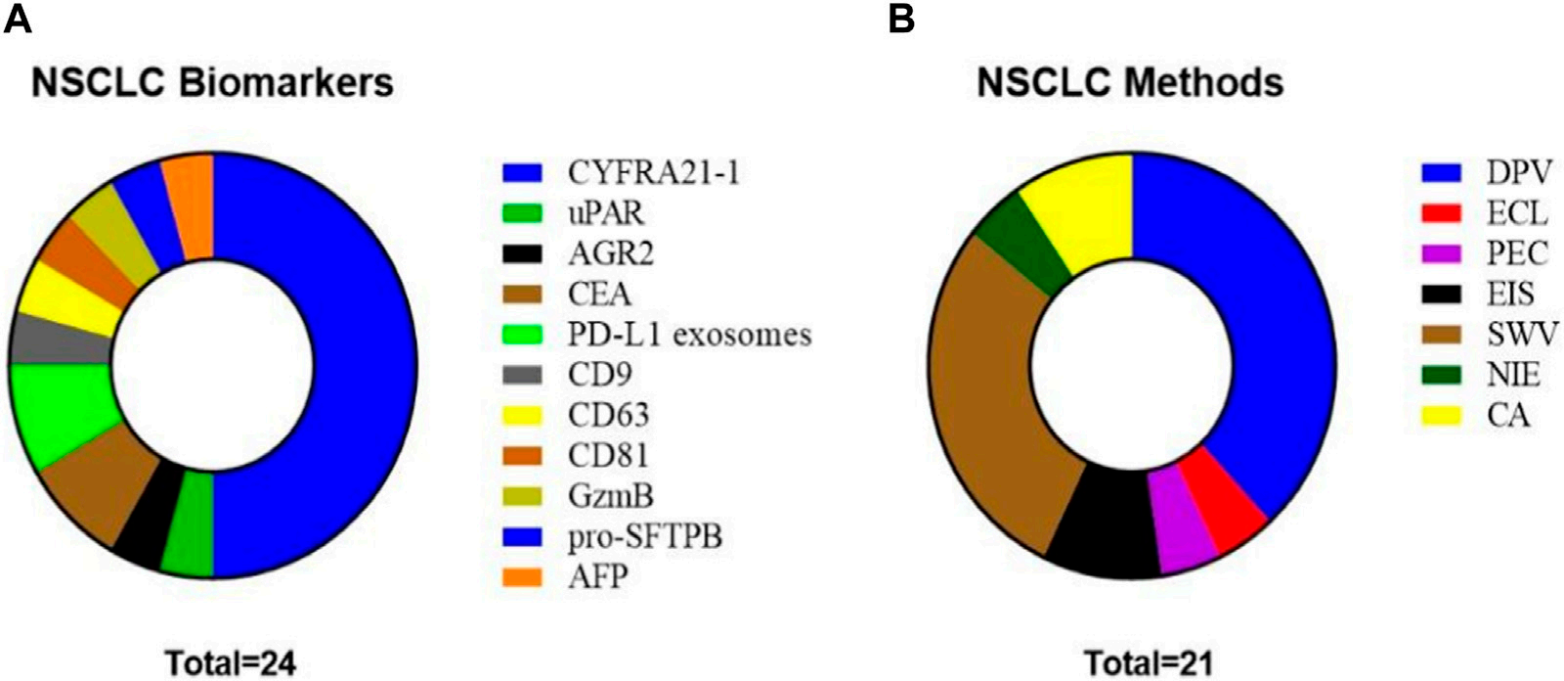
**(A)** NSCLC biomarkers studied in the reviewed articles. **(B)** Methods used in the reviewed articles for biomarker analysis in NSCLC.

NSCLC is the most common form of lung cancer, accounting for about 85% of lung cancer cases. Because early detection and treatment of LC patients are closely related to an increased survival rate, accurate screening methods are needed. Some biomarkers for this specific cancer type have been analyzed in the last 5 years using electrochemical detection methodologies such as CYFRA21-1, anterior gradient-2 protein (AGR2), CEA, programmed dead-ligand 1 (PD-L1), protein member of the transmembrane 4 superfamily (CD9), protein associated with membranes of intracellular vesicles (CD63), cluster of differentiation 81 (CD81), urokinase-type plasminogen activator receptor (uPAR), granzyme B (GzmB), pro-surfactant protein B (pro-SFTPB), and AFP. Figure 6 depicts the distribution of biomarkers and electrochemical methods used by the different authors. CYFRA21-1 is the most detected biomarker, and DPV is the most popular technique, followed by square wave voltammetry (SWV).

#### 4.2.1 CYFRA21-1

CYFRA21-1 has been extensively evaluated as an NSCLC biomarker and, in consequence, many sensors have been developed for its analysis. It is part of the cytokeratins family and forms the cytoskeleton filaments of all epithelial cells. Its application in LC diagnosis is very well documented, with a diagnosis cut-off of 3.5 ng/mL (Okamura et al., 2013). In 2018, Zeng et al. (2018) reported a sandwich-type electrochemical immunosensor showcasing a composite structure consisting of three-dimensional graphene (3D-G), chitosan (CS), and glutaraldehyde (GA) on a GCE to expand the surface area with exceptional conductivity. Then, in another interesting approach, Meng et al. (2019) presented an electrochemiluminescent (ECL) immunoassay by using molybdenum oxide quantum dots (MoOx QDs). Later, Feng et al. (2021) presented a microfluidic system that combined a microelectrode and a cathodic photoelectrochemical (PEC) biosensor. The amplification mechanism hinges on the p–n junction of AgI/Bi_2_Ga_4_O_9_, with dissolved O_2_ serving as an electron acceptor, resulting in the production of superoxide anion radicals (˙O_2_−) as the working microelectrode. To further enhance the detection capability, a novel superoxide-dismutase-loaded honeycomb manganese oxide nanostructure (SOD@hMnO_2_) was employed as a co-catalyst signal amplification label.

Yola et al. (2021) developed a sensor that employs a composite material comprised of silicon nitride (Si_3_N_4_) and molybdenum disulfide (MoS_2_) incorporated onto multi-walled carbon nanotubes (MWCNTs) as the reaction platform. Additionally, it utilizes core-shell magnetic mesoporous silica nanoparticles@gold nanoparticles (MMSNs@AuNPs) as a signal amplifier. In a study by Yang et al. (2021) that aimed to detect CYFRA21-1, they distributed GNPs on the surface of three-dimensional graphene (3D-G) attached to a GCE with thionine (thi) and m-cresol purple (MCP) as signal tags. In the same year, Wang et al. (2021) presented an electrochemical immunosensor where graphene oxide (GO) was immobilized into the GCE as an immune reaction support. Then, a secondary antibody was conjugated with 4-cyano-4-(phenylcarbonothioylthio) pentanoic acid (CPAD) to be introduced into the sandwich reaction. Finally, the immunocomplex was linked to numerous monomers through reversible addition-fragmentation chain transfer (RAFT) polymerization. In another work, Lu et al. (2021) proposed a novel electrochemical immunosensor based on the ring-opening polymerization (ROP) signal amplification strategy. At the same time, Cheng and Hu. (2021) developed a sandwich-type electrochemical immunosensor based on a combination of gold nanoparticle (AuNPs) decorated Ti_3_C2Tx-MXene (Au-Ti_3_C2Tx) over a GCE. Another novel sensor proposed by Zhang J. et al. (2021) employed nanoparticle impact electrochemistry (NIE) using silver nanoparticles (AgNPs) as labels. The approach is grounded in measuring the impact frequency and the charge intensity resulting from the electrochemical oxidation of individual AgNPs. Recently, Gu et al. (2022) chose mesoporous carbon CMK-3 to mix with carboxylated multi-walled carbon nanotubes (CMWCNTs) to enhance electron transfer efficiency. Furthermore, gold nanoparticles (AuNPs) were incorporated to offer a multitude of binding sites for antibodies.

In another interesting approach, Feng et al. (2022) developed an immunosensor by encapsulating an electroactive dye, such as methylene blue (MB), to enhance signal amplification. In the same year, Hu et al. (2022) synthesized AuNPs@MoS_2_@Ti_3_C_2_Tx composites, where titanium carbide (Ti_3_C_2_Tx) was modified with gold nanoparticles (AuNPs) and molybdenum disulfide (MoS_2_) and used as a solid support. Finally, Jafari-Kashi et al. (2022) designed a novel label-free electrochemical DNA biosensor based on the oxidation signal of guanine.

#### 4.2.2 NO CYFRA21-1 biomarkers

**FIGURE 7:**
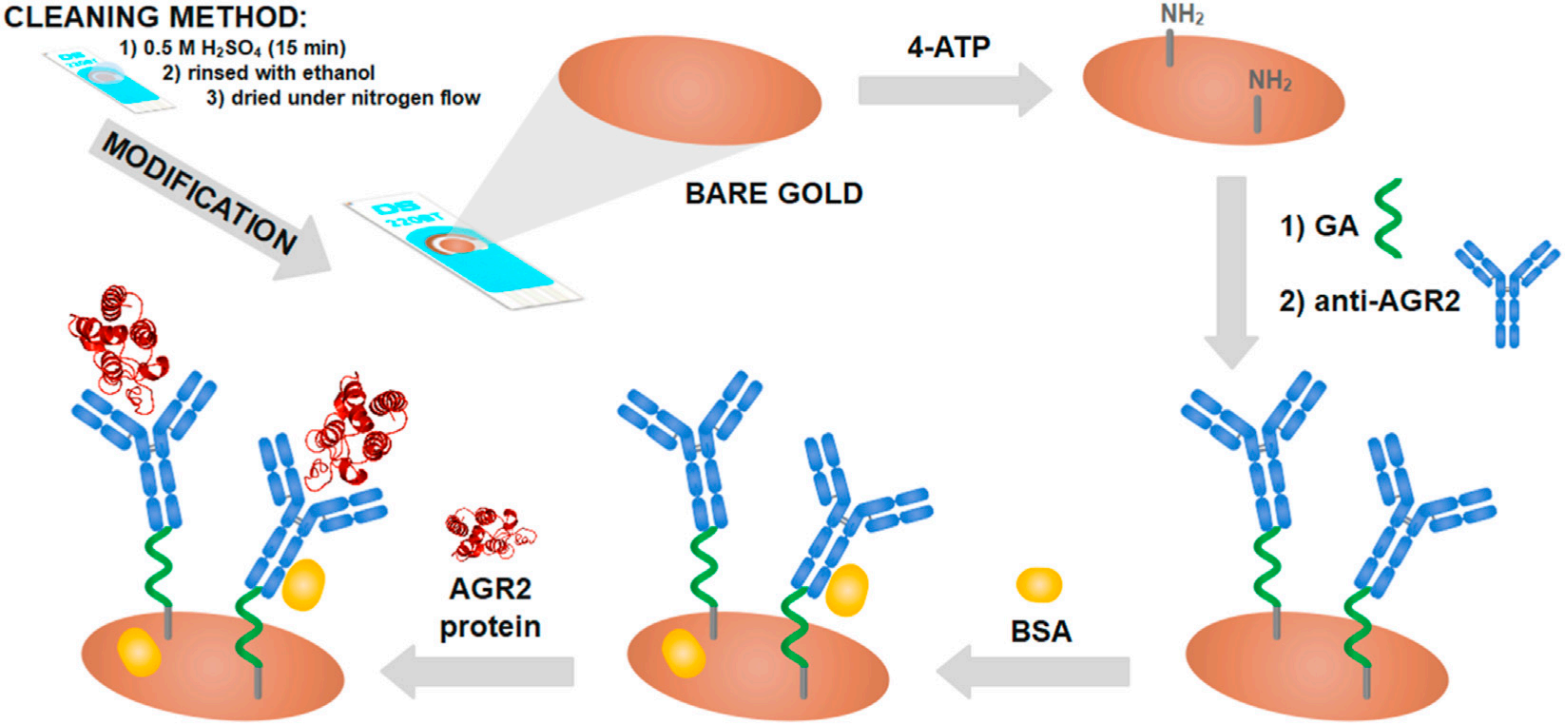
Schematic stages of modified screen-printed gold electrodes. 4-ATP is 4-aminothiophenol, GA is glutaraldehyde, and anti-AGR2 is the AGR2 antibody. Reprinted with permission from Białobrzeska et al. (2021) (https://creativecommons.org/licenses/by/4.0/).

In recent years, an increasing number of promising biomarkers with clinical relevance in NSLC have been discovered, and several electrochemical analysis developments have resulted. In this sense, Roberts et al. (2019) developed an electrochemical immunosensor for the swift identification of urokinase-type plasminogen activator receptor (uPAR), an important factor of the fibrinolytic system in tumor progression and metastasis. This investigation employed a cutting-edge ultrasensitive electrode crafted from graphene nanosheets (GNS) functionalized with a monoclonal antibody. Next, Białobrzeska et al. (2021) fabricated a biosensor based on a screen-printed gold electrode functionalized with monoclonal antibodies for impedance quantification of anterior gradient-2 protein (AGR2) that is a protein widely secreted in precancerous lesions, primary tumors, and metastatic tumors (Figure 7). Saputra et al. (2022) developed an amperometric biosensor for the sensitive detection of granzyme B (GzmB) using a dual monomer-based bioconjugate; GzmB is a serine protease highly expressed in immune cells and tumor microenvironments that is a promising biomarker when it is detected in serum. Furthermore, Chai et al. (2022) developed an electrochemical immunosensor for the ultrasensitive detection of pro-surfactant protein B (pro-SFTPB). This proprotein is produced by alveolar cells and released into the alveolar compartments. This sensor utilized innovative binary PdCu mesoporous metal nanoparticles as signal labels and employed black phosphorus (BP) nanosheets and AuNPs for electrode modification. Finally, Taheri et al. (2022) developed a highly effective sensing layer using a dual-template molecularly imprinted polymer (DMIP) for the individual detection of two lung cancer biomarkers: CEA and AFP.

#### 4.2.3 Extracellular vesicles

Interestingly, three manuscripts focused on the quantification of specific exosomes with clinical relevance in NSCLC. Wang M. et al. (2022) developed a highly sensitive biosensor capable of detecting programmed dead-ligand 1 positive (PD-L1+) exosomes by utilizing covalent organic framework nanosheets (2D COF NSs) and CRISPR-Cas12a for signal amplification. Then, Sha et al. (2022) presented a DNA-fueled electrochemical sensor for the identification of PD-L1+ exosomes. These exosomes were enriched by using magnetic beads decorated with PD-L1 antibodies and could interact with cholesterol-modified hairpin templates. Then, programmable DNA synthesis starting from the hairpin template triggers a primer exchange reaction and generates a large number of extension products to activate the trans-cleavage activity of CRISPR-Cas12a to enhance the reaction. Finally, Zhang Y. et al. (2022) developed an MOF-based electrochemical liquid biopsy (ELB) platform that offers efficient, sensitive, and multiplexed detection of cancer exosomes by detecting CD9, CD63, and CD81, which are the members of the tetraspanin family employed as an EV-specific marker. Table 3 summarizes the principal characteristics of reviewed articles about NSCLC.

**TABLE 3:** Summarize the principal characteristics of reviewed articles about NSCLC.

Reference	Biomarker	Method	Redox signaling species	Modification	Recognition	LOD	Linear range	Sample
Zeng et al. (2018)	CYFRA21-1	DPV	[Fe(CN)_6_]^3−/4−^	Ab1-CYFRA21-1/3D–G/GCE	Antibodies	43 pg/mL	0.1–150 ng/mL	Serum
				HRP-Ab2/AuNPs/Thi/MWCNT-NH_2_				
Meng et al. (2019)	CYFRA21-1	ECL	MoOx QDs/S_2_O_8_^2−^	GCE/MoOx QDs/Au NP-CS/Anti-CYFRA21-1	Antibodies	0.3 pg/mL	1 pg/mL–350 ng/mL	Serum
Roberts et al. (2019)	uPAR	DPV	[Fe(CN)_6_]^3−/4−^	FTO-GNS/uPAR-Ab	Antibodies	4.8 fM	1 fM–1 μM	Serum
Feng et al. (2021)	CYFRA21-1	PEC	[Fe(CN)_6_]^3−/4−^	Ab1/p–n AgI/Bi_2_Ga_4_O_9_/ITO	Antibodies	0.026 pg/mL	0.1 pg/mL–100 ng/mL	Serum
				SOD@hMnO_2_–Ab2				
Yola et al. (2021)	CYFRA21-1	DPV	[Fe(CN)_6_]^3−/4−^	Anti-CYFRA21-1-Ab1/Si_3_N_4_/MoS_2_–MWCNTs/GCE	Antibodies	2 fg/mL	0.01–1.0 pg/mL	plasma
				MMSNs@AuNPs/anti-CYFRA21-1-Ab2				
Białobrzeska et al. (2021)	AGR2	EIS	[Fe(CN)_6_]^3−/4−^	GSPE	Antibodies	0.093 fg/mL	0.01 fg/mL–10 fg/mL	Cell lysate
Yang et al. (2021)	CEA	SWV	[Fe(CN)_6_]^3−/4−^	Anti-CEA/anti-CYFRA21-1/3D-G/AuNPs/GCE	Antibodies	0.31	0.5–200 ng/mL	Serum
	CYFRA21-1			Au-pThi-anti-CYFRA21-1		0.18 ng/mL		
				Au-pMCP-anti-CEA				
Wang et al. (2021)	CYFRA21-1	SWV	[Fe(CN)_6_]^3−/4−^	Ab1/GO/GCE	Antibodies	0.14 fg/mL	0.5 fg/mL–10 pg/mL	Serum
				CPAD-Ab2				
Lu et al. (2021)	CYFRA21-1	DPV	[Fe(CN)_6_]^3−/4−^	GE/anti-CYFRA21-1-Ab1	Antibodies	9.08 fg/mL	1 pg/mL–1 mg/mL	Serum
				dAb/NCA				
Cheng and Hu. (2021)	CYFRA21-1	SWV	TB	Ab1/AuNPs/Ti_3_C_2_Tx/GCE	Antibodies	0.1 pg/mL	0.5–1×10^4^ pg/mL	Serum
				Ab2/TB-Au-COF				
Zhang et al. (2021a)	CYFRA21-1	NIE	[Fe(CN)_6_]^3−/4−^	CFM/AgNPs/Ab1	Antibodies	0.1 ng/mL	0.1–10 ng/mL	Serum
Wang et al. (2022a)	PD-L1 exosomes	SWV	[Fe(CN)_6_]^3−/4−^	MB-aptamer DNA (PD-L1 aptamer)/AuNPs@COFNSs/GCE	Aptamer	38 particles/μL	1.2×10^2^–1.2×10^7^ particles/μL	Serum
Gu et al. (2022)	CYFRA21-1	DPV	[Fe(CN)_6_]^3−/4−^	AuNPs@CMK-3@CMWCNTs/GCE	Antibodies	0.2 pg/mL	0.5 pg/mL–10^5^ pg/mL	Serum
Feng et al. (2022)	CYFRA21-1	DPV	MB	Au@Rh DNCs	Antibodies	31.72 fg/mL	100 fg/mL–100 ng/mL	Serum
				MB/PtPd/PDA/HCSs				
Zhang et al. (2022a)	CD9	DPV	Ag^0^(Red)/Ag^+^(Ox)	ZIF-90-ZnO-MoS_2_/Carbon ink	Antibodies	10	5 × 10^4^–1 × 10^−5^ ng/mL	Cell supernatant - Blood
	CD63					1		
	CD81					1 fg/mL		
	Exosomes							
Jafari-Kashi et al. (2022)	CYFRA21-1	DPV	[Fe(CN)_6_]^3−/4−^	GCE/rGO/PPy/AgNPs/ssDNA	DNA	2.4 fM	1 × 10^−14^–1 × 10^−6^ M	PBS
Saputra et al. (2022)	GzmB	CA	BCB	SPCE/AuNPs/pPATT-Ab1	Antibodies	1.75 pg/mL	3.0–50.0	Serum
				AuNPs/pPATT/TBA/BCB/Ab2			50.0–1,000 pg/mL	
Chai et al. (2022)	pro-SFTPB	CA	H_2_O_2_	Ab1/AuNPs/BP nanosheets/GCE	Antibodies	5.3 pg/mL	10 pg/mL−100 ng/mL	Serum
				Ab2/PdCu MMN				
Hu et al. (2022)	CYFRA21-1	SWV	TB	GCE/Nafion-AuNPs/Ab1	Antibodies	0.03 pg/mL	0.5 pg/mL–50 ng/mL	Serum
				AuNPs@MoS_2_@Ti_3_C_2_Tx/TB-Ab2				
Sha et al. (2022)	PD-L1 exosomes	SWV	MB	GE/PDDA/AuNPs/CB	Antibodies	708 particles/mL	10^3^–10^9^ particles/mL	Serum
Taheri et al. (2022)	CEA	EIS	[Fe(CN)_6_]^3−/4−^	PPy-MO/FTO	MIP	1.6	5–10^4^	Serum
	AFP					3.3 pg/mL	10–10^4^ pg/mL	

CA, chronoamperometry; DPV, differential pulse voltammetry; ECL, electrogenerated chemiluminescence; EIS, electrochemical impedance spectroscopy; SWV, square wave voltammetry; NIE, nanoparticle impact electrochemistry; PEC, photoelectrochemical; CYFRA21.1, cytokeratin fragment; uPAR, urokinase-type plasminogen activator receptor; AFP, alpha-fetoprotein; AGR2, anterior gradient 2; CEA, carcinoembryonic antigen; PDL1, programmed death-ligand 1; CD9, protein member of the transmembrane 4 superfamily; CD63, protein associated with membranes of intracellular vesicles; CD81, cluster of differentiation 81; GzmB, granzyme B; SFTPB, pulmonary-surfactant protein; MoOx QDs/S_2_O_8_^2−^, molybdenum oxide quantum dots/S_2_O_8_^2−^; TB, toluidine blue; MB, methylene blue; BCB, brilliant cresyl blue; Ab1-CYFRA21-1/3D–G/GCE, primary antibody-CYFRA21-1/three-dimensional graphene/glassy carbon electrode; HRP-Ab2/AuNPs/Thi/MWCNT-NH_2_, horseradish peroxidase-secondary antidoby/gold nanoparticles/thionine/amino-functionalized carbon nanotube; GCE/MoOx QDs/AuNP-CS/Anti-CYFRA21-1, glassy carbon electrode/molybdenum oxide quantum dots/gold nanoparticles-CS/CYFRA21-1 primary antibody; FTO-GNS/uPAR-Ab, fluorine-doped tin oxide- graphene nanosheet/urokinase-type plasminogen activator receptor; Ab1/p–n AgI/Bi_2_Ga_4_O_9_/ITO, antibody primary/p–n junction AgI/Bi_2_Ga_4_O_9_/indium tin oxide; SOD@hMnO_2_–Ab2, superoxide dismutase@ hMnO_2_–secondary antibody; anti-CYFRA21-1-Ab1/Si_3_N_4_/MoS_2_–MWCNTs/GCE, CYFRA21-1 antibody-primary antibody/Si_3_N_4_/MoS_2_–multi-wall carbon nanotubes/glassy carbon electrode; MMSNs@AuNPs/anti-CYFRA21-1-Ab2, magnetic mesoporous silica nanoparticles@gold nanoparticles/CYFRA21-1 antibody-secondary antibody; GSPE, gold screen-printed electrodes; anti-CEA/anti-CYFRA21-1/3D-G/AuNPs/GCE, CEA primary antibody/CYFRA21-1 primary antibody/three-dimensional graphene/gold nanoparticles/glassy carbon electrode; Au-pThi-anti-CYFRA21-1, Au-poly-thionine- CYFRA21-1 primary antibody; Au-pMCP-anti-CEA, Au-poly-m-cresol purple-CEA antibody; Ab1/GO/GCE, primary antibody/graphene oxide/glassy carbon electrode; CPAD-Ab2, secondary antibody; GE/anti-CYFRA21-1-Ab1, gold electrode/CYFRA21-1 antibody-primary antibody; dAb/NCA, detection antibody/N-carboxyanhydride; Ab1/AuNPs/Ti_3_C_2_Tx/GCE, primary antibody/gold nanoparticles/transition metal two-dimensional carbides, nitrides, and carbonitrides/glassy carbon electrode; Ab2/TB-Au-COF, secondary antibody/TB-Au- covalent organic framework polymers; CFM/AgNPs/Ab1, carbon fiber microelectrode/silver nanoparticles/primary antibody; MB-aptamer DNA (PD-L1 aptamer)/AuNPs@COFNSs/GCE, MB-aptamer DNA (programmed death-ligand 1 aptamer)/gold nanoparticles@ covalent organic frameworks nanosheets/glassy carbon electrode; AuNPs@CMK-3@CMWCNTs/GCE, gold nanoparticles@ordered mesoporous carbon @carboxylated multi-walled carbon nanotubes/glassy carbon electrode; Au@Rh DNCs, Au@Rh dendritic nanocrystals; MB/PtPd/PDA/HCSs, MB/PtPd nanoparticles/polydopamine/carbon spheres; ZIF-90-ZnO-MoS_2_/carbon ink, metal–organic framework (MOF) ZIF-90-ZnO-MoS2 nanohybrid/carbon ink; GCE/rGO/PPy/AgNPs/ssDNA, glassy carbon electrode/reduced graphene oxide/poly pyrrole/silver nanoparticles/single-strand DNA; SPCE/AuNPs/pPATT-Ab1, screen-printed carbon electrode/gold nanoparticles/poly3′-(2-aminopyrimidyl)- 2,2′:5′,2″-terthiophene/primary antibody; AuNPs/pPATT/TBA/BCB/Ab2, gold nanoparticles/poly3′-(2-aminopyrimidyl) 2,2′:5′,2″-terthiophene/2,2:5,2-terthiophene-3-(p-benzoic acid)/brilliant cresyl blue/secondary antibody; Ab1/AuNPs/BP nanosheets/GCE, primary antibody/BP nanosheets/glassy carbon electrode; Ab2/PdCu MMN, secondary antibody/PdCu mesoporous metal nanoparticles; GCE/Nafion-AuNPs/Ab1, glassy carbon electrode/Nafion-gold nanoparticles/primary antibody; AuNPs@MoS_2_@Ti_3_C_2_Tx/TB-Ab2, gold nanoparticles/MoS_2_/transition metal two-dimensional carbides, nitrides, and carbonitrides/toluidine blue-secondary antibody; GE/PDDA/AuNPs/CB, graphite electrode/poly-(diallyldimethylammonium chloride)/gold nanoparticles/cucurbit uril; PPy-MO/FTO, electropolymerized polypyrrole-methyl orange/fluorine-doped tin oxide.

### 4.3 Small cell lung cancer (SCLC)

**FIGURE 8:**
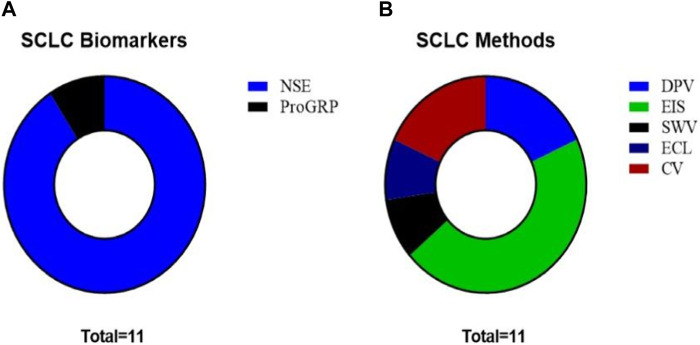
**(A)** SCLC biomarkers studied in the reviewed articles. **(B)** Methods employed in the reviewed articles for biomarker analysis in SCLC.

Approximately 15%–20% of the new cases of LC are small cell lung cancer; these cancers are at an advanced stage by the time most of these patients are diagnosed. In the last 5 years, most manuscripts analyzed the NSE biomarker; this protein is a glycolytic enzyme largely expressed in neuroendocrine tumors. NSE can serve as the assumed serum or plasma marker of this cancer and exhibits a remarkably diagnostic specificity and sensitivity. Its application as an SCLC biomarker is being employed with a standardized cut-off of 20.5 ng/mL (Satoh et al., 2002). In this section, we review the articles focused on this tumor type (Figure 8).

**FIGURE 9:**
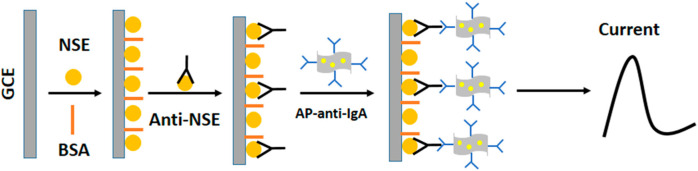
Schematic representation of the designed electrochemical immunosensor for neuron-specific enolase (NSE) detection. Reprinted with permission from Wei et al. (2017) (https://creativecommons.org/licenses/by/4.0/).

Ten studies focused on the analysis of NSE. Among these, Wei et al. (2017) developed an electrochemical immunosensor using a gold nanoparticle/reduced graphene oxide composite (AuNP-RGO) (Figure 9). Then, Zhang et al. (2018) reported a label-free electrochemical immunoassay using a three-dimensionally macroporous reduced graphene oxide/polyaniline (3DM rGO/PANI) film. In the next year, Aydin et al. (2019) developed an impedimetric immunosensor based on a poly(thiophene)-graft-poly(methacrylamide) polymer [P(Thi-g-MAm)]-modified ITO electrode for neuron-specific enolase detection in human serum. The P(Thi-g-MAm) polymer acts as a platform for the immobilization of NSE-specific monoclonal antibodies. In the same year, Fang et al. (2019) designed an electrochemical immunoassay with a triple signal amplification strategy using a porous three-dimensional graphene-starch architecture (3D-GNS). In addition, Aydin et al. (2020) designed a label-free immunosensor for NSE detection using an epoxy-substituted-polypyrrole P(Pyr-Epx) polymer as an immobilization platform. Furthermore, Yi et al. (2022) constructed an electrochemical immunosensor based on a reduced graphene oxide/Cu_8_Ni_2_ (rGO/Cu_8_Ni_2_) nanocomposite. In that year, Li et al. (2022) developed an electrochemiluminescence biosensor using CePO_4_/CeO_2_ heterostructures as sensing substrates.

Karaman et al. (2022) developed an electrochemical immunosensor using gold nanoparticle-modified molybdenum disulfide and reduced graphene oxide (AuNPs@MoS_2_/rGO) as the electrode platform. Khatri and Puri (2022a), Khatri and Puri (2022b) presented two electrochemical sensors in 2022. The first electrochemical biosensor for NSE detection used rGO-modified r-MoS2 multi-layered nanosheets as a matrix, and the second one was an immunosensor that used gold nanoparticle-modified molybdenum disulfide and reduced graphene oxide (AuNPs@MoS_2_/rGO) as the electrode platform. Lastly, Liu et al. (2021) reported an electrochemical immunosensor for the detection of a singular biomarker called pro-gastrin-releasing peptide (ProGRP). This sensor was fabricated based on a three-dimensional reduced graphene oxide-gold nanoparticles (3D-rGO@Au) composite. Table 4 summarizes the principal characteristics of reviewed articles about SCLC.

**TABLE 4:** Summarize the principal characteristics of reviewed articles about SCLC.

Reference	Biomarker	Technique	Redox signaling species	Modification	Recognition	LOD	Linear range	Sample
Wei et al. (2017)	NSE	DPV	[Fe(CN)_6_]^3−/4−^	AuNP-RGO	Antibodies	0.05 ng/mL	0.1–2,000 ng/mL	Serum
Zhang et al. (2018)	NSE	DPV	[Fe(CN)_6_]^3−/4−^	3DM rGO/PANI	Antibodies	0.05 ng/mL	0.5 pg/mL–10.0 ng/mL	Serum
Aydın et al. (2019)	NSE	EIS	[Fe(CN)_6_]^3−/4−^	P(Thi-g-MAm) polymer	Antibodies	6.1 fg/mL	0.02–4 pg/mL	Serum
Fang et al. (2019)	NSE	ASV	KCl	3D-GNS	Antibodies	0.008 pg/mL	0.02 pg/mL–35 ng/mL	Serum
Aydın et al. (2020)	NSE	EIS	[Fe(CN)_6_]^3−/4−^	P(Pyr-Epx)	Antibodies	6.1 fg/mL	0.02–7.5 pg/mL	Serum
Liu et al. (2021)	ProGRP	SWV	[Fe(CN)_6_]^3−/4−^	3D-rGO@Au	Antibodies	0.14 fg/mL	1 fg/mL–10 ng/mL	Serum
Yi et al. (2022)	NSE	EIS	[Fe(CN)_6_]^3−/4−^	rGO/Cu_8_Ni_2_	Antibodies	137 fg/mL	500 fg/mL–50 ng/mL	Serum
Li et al. (2022)	NSE	ECL	Luminol–H_2_O_2_	CePO_4_/CeO_2_	Antibodies	72.4 fg/mL	76 fg/mL–100 ng/mL	Serum
Khatri and Puri (2022a)	NSE	EIS	[Fe(CN)_6_]^3−/4−^	rGO-modified r-MoS_2_	Antibodies	1 ng/mL	1–200 ng/mL	Serum
		CV				2.32 ng/mL	0.1–100 ng/mL	
Khatri and Puri (2022b)	NSE	CV	[Fe(CN)_6_]^3−/4−^	CS/MoS_2_	Antibodies	3.35 ng/mL	0.1–100 ng/mL	Serum
Karaman et al. (2022)	NSE	EIS	[Fe(CN)_6_]^3−/4−^	AuNPs@MoS_2_/rGO	Antibodies	3 fg/mL	0.01–1 pg/mL	Plasma

CV, cyclic voltammetry; DPV, differential pulse voltammetry; ECL, electrogenerated chemiluminescence; EIS, electrochemical impedance spectroscopy; SWV, square wave voltammetry; ASV, anodic stripping voltammetry; NSE, neuron-specific enolase; ProGRP, pro-gastrin-releasing peptide; Luminol-H_2_O_2_, luminol–hydrogen peroxide solution; AuNP-RGO, gold nanoparticles-reduced graphene oxide; 3DM rGO/PANI, three-dimensionally macroporous reduced graphene oxide/polyaniline; P(Thi-g-MAm) polymer, poly(thiophene)-graft-poly(methacrylamide) polymer; 3D-GNS, porous three-dimensional graphene-starch architecture; P(Pyr-Epx), epoxy-substituted-polypyrrole; 3D-rGO@Au, three-dimensional reduced graphene oxide-gold nanoparticles; rGO/Cu_8_Ni_2_, reduced graphene oxide/Cu_8_Ni_2_; CePO_4_/CeO_2_, heterostructures; rGO-modified r-MoS_2_, reduced graphene oxide-modified r-MoS_2_; CS/MoS_2_, chitosan/MoS_2_; AuNPs@MoS_2_/rGO, gold nanoparticles@MoS_2_/reduced graphene oxide.

## 5 Discussion

The present review has identified the application of electrochemical detection methodologies into three sections: Total LC, NSCLC, and SCLC. In total LC, the focus is on various biomarkers such as SOX-2, AFP, CEA, NSE, Cyfra21-1, CD44, EGFR, and others. The review highlights studies utilizing several highly sensitive electrochemical techniques and electrode modifications for improved analytical properties in real clinical samples. The examples provided, like the electrochemical detection of SOX-2 and CEA, showcase the diversity in methods, including microfluidic devices and biosensors, to enhance sensitivity and specificity through the use of biomolecules such as monoclonal antibodies and aptamers.

The discussion on NSCLC emphasizes the analysis of biomarkers such as CYFRA21-1, AGR2, CEA, PD-L1, CD9, CD63, CD81, Upar, GzmB, pro-SFTPB, and AFP. The graphical representation in Figure 4 illustrates the distribution of biomarkers and the popularity of electrochemical techniques such as differential pulse voltammetry (DPV) and square wave voltammetry (SWV). The prominence of CYFRA21-1 as the most detected biomarker suggests its potential significance in NSCLC diagnosis.

For SCLC, which accounts for 15%–20% of LC cases, the focus is primarily on the NSE biomarker. NSE is highlighted for its diagnostic specificity and sensitivity. The discussion in this section may provide insights into the potential of NSE as a marker for SCLC, offering a basis for future research and clinical applications.

At the same time, it is important to discuss the rapid advancement in biosensor development for clinical biomarker analysis. Examples include the many sensors developed for biomarkers whose clinical applications have yet to be demonstrated or do not yet have standardized cut-off values. Only CEA, CYFRA-21, and NSE have predefined values for diagnosis and are the only markers widely investigated in clinical settings. The appearance of a new promising biomarker typically leads to the development of an electrochemical sensor that, in principle, meets the analytical requirements of the biomarker, even when those values have not been well studied. This suggests that, in lung cancer, there is no time to wait for the true value of the analyte before initiating significant efforts to develop new analytical strategies. However, these efforts could be in vain if those biomarkers do not demonstrate good performance and applicability. In the near future, considering the need for new biomarkers with diagnostic and predictive value and the increasing research in biomarker discovery, the number of electrochemical sensors in all subtypes of lung cancer will continue to increase in the same way as we have observed over the last 5 years.

## 6 Conclusion

This review provides a comprehensive overview of recent advancements in electrochemical detection methodologies for LC biomarkers. The inclusion of specific studies, methodologies, and detection limits contributes to the depth of the discussion, making it a valuable resource for researchers and clinicians interested in the field of LC diagnosis. Specific LC biomarkers can be detected with modern electrochemical methodologies and detection techniques. Methodologies based on the development of electrochemical immunosensors and microfluidic devices with the incorporation of nanomaterials as platforms for immobilizing biomolecules, such as specific monoclonal antibodies, are widely used in cancer diagnosis. In addition, multiplex electrochemical devices have been developed that have the advantage of detecting more than one specific biomarker at a time. In general, these electrochemical methodologies present advantages such as short analysis times, low cost, portability, low detection limits, and low consumption of reagents and samples. In addition, qualified personnel are not needed to use these methodologies. Finally, methodologies based on electrochemical detection techniques represent an interesting analytical tool that can be of great help for LC diagnosis and monitoring of the treatment of the disease in patients.

## References

[B1] Aydın E. B. Aydın M. Sezgintürk M. K. (2020). Selective and ultrasensitive electrochemical immunosensing of NSE cancer biomarker in human serum using epoxy-substituted poly(pyrrole) polymer modified disposable ITO electrode. Sensors Actuators, B Chem. 306, 127613. 10.1016/j.snb.2019.127613

[B2] Aydın E. B. Sezgintürk M. K. (2017). A sensitive and disposable electrochemical immunosensor for detection of SOX2, a biomarker of cancer. Talanta 172, 162–170. 10.1016/j.talanta.2017.05.048 28602290

[B3] Aydın M. Aydın E. B. Sezgintürk M. K. (2019). A highly selective poly(thiophene)-graft-poly(methacrylamide) polymer modified ITO electrode for neuron-specific enolase detection in human serum. Macromol. Biosci. 19, e1900109–e1900112. 10.1002/mabi.201900109 31222894

[B4] Białobrzeska W. Dziąbowska K. Lisowska M. Mohtar M. A. Muller P. Vojtesek B. (2021). An ultrasensitive biosensor for detection of femtogram levels of the cancer antigen agr2 using monoclonal antibody modified screen-printed gold electrodes. Biosensors 11, 184–211. 10.3390/bios11060184 34200338 PMC8230265

[B5] Brett C. Brett A. (1993). Electrochemistry: principles, methods, and applications. New York: Oxford University Press.

[B6] Chai X. Gao J. Cui Q. Zhao L. (2022). Enzyme-free sandwich-type electrochemical immunosensor based on high catalytic binary PdCu mesoporous metal nanoparticles and conductive black phosphorous nanosheets for ultrasensitive detection of pro-SFTPB in non-small cell lung cancer. J. Electroanal. Chem. 917, 116415. 10.1016/j.jelechem.2022.116415

[B7] Chen Y. Sun L. Qiao X. Zhang Y. Li Y. Ma F. (2020). Signal-off/on electrogenerated chemiluminescence deoxyribosensors for assay of early lung cancer biomarker (NAP2) based on target-caused DNA charge transfer. Anal. Chim. Acta 1103, 67–74. 10.1016/j.aca.2019.12.049 32081190

[B8] Cheng J. Hu K. Liu Q. Liu Y. Yang H. Kong J. (2021). Electrochemical ultrasensitive detection of CYFRA21-1 using Ti_3_C_2_T_x_-MXene as enhancer and covalent organic frameworks as labels. Anal. Bioanal. Chem. 413, 2543–2551. 10.1007/S00216-021-03212-Y 33576855

[B9] Deepa B. Chaudhary R. Pundir C. S. (2022). Amperometric detection of tumor suppressor protein p53 via pencil graphite electrode for fast cancer diagnosis: p53 targeted immunosensor for early diagnosis of cancer. Anal. Biochem. 639, 114528. 10.1016/j.ab.2021.114528 34919898

[B10] Fan C. Jiang B. Shi W. Chen D. Zhou M. (2022). Tri‐Channel electrochemical immunobiosensor for combined detections of multiple exosome biomarkers of lung cancer. Biosensors 12, 435. 10.3390/bios12070435 35884238 PMC9313016

[B11] Fang Y. Li Y. Zhang M. Cui B. Hu Q. Wang L. (2019). A novel electrochemical strategy based on porous 3D graphene-starch architecture and silver deposition for ultrasensitive detection of neuron-specific enolase. Analyst 144, 2186–2194. 10.1039/c8an02230e 30785140

[B12] Feng J. Wu T. Cheng Q. Ma H. Ren X. Wang X. (2021). A microfluidic cathodic photoelectrochemical biosensor chip for the targeted detection of cytokeratin 19 fragments 21-1. Lab a Chip 21, 378–384. 10.1039/d0lc01063d 33313636

[B13] Feng Y. G. He J. W. Chen D. N. Jiang L. Y. Wang A. J. Bao N. (2022). A sandwich-type electrochemical immunosensor for CYFRA 21–1 based on probe-confined in PtPd/polydopamine/hollow carbon spheres coupled with dendritic Au@Rh nanocrystals. Microchim. Acta 189, 271–279. 10.1007/s00604-022-05372-9 35789294

[B14] Grunnet M. Sorensen J. B. (2012). Carcinoembryonic antigen (CEA) as tumor marker in lung cancer. Lung Cancer 76, 138–143. 10.1016/j.lungcan.2011.11.012 22153832

[B15] Gu Y. Jiang Y. Gong G. Cheng X. Mei Y. Pan H. (2022). Detection of CYFRA21-1 in serum by electrochemical immunosensor based on nanocomposite consisting of AuNPs@CMK-3@CMWCNTs. Bioelectrochemistry 148, 108230. 10.1016/j.bioelechem.2022.108230 36029760

[B16] Hu K. Cheng J. Wang K. Zhao Y. Liu Y. Yang H. (2022). Sensitive electrochemical immunosensor for CYFRA21-1 detection based on AuNPs@MoS2@Ti3C2T composites. Talanta 238, 122987. 10.1016/j.talanta.2021.122987 34857321

[B17] Huang Z. Xu D. Zhang F. Ying Y. Song L. (2016). Pro-gastrin-releasing peptide and neuron-specific enolase: useful predictors of response to chemotherapy and survival in patients with small cell lung cancer. Clin. Transl. Oncol. 18, 1019–1025. 10.1007/s12094-015-1479-4 26886220

[B18] Jafari-Kashi A. Rafiee-Pour H. A. Shabani-Nooshabadi M. (2022). A new strategy to design label-free electrochemical biosensor for ultrasensitive diagnosis of CYFRA 21–1 as a biomarker for detection of non-small cell lung cancer. Chemosphere 301, 134636. 10.1016/j.chemosphere.2022.134636 35447211

[B19] Karachaliou N. Rosell R. Viteri S. (2013). The role of SOX2 in small cell lung cancer, lung adenocarcinoma and squamous cell carcinoma of the lung. Transl. Lung Cancer Res. 2, 172–179. 10.3978/j.issn.2218-6751.2013.01.01 25806230 PMC4367598

[B20] Karaman C. Bölükbaşı Ö. S. Yola B. B. Karaman O. Atar N. Yola M. L. (2022). Electrochemical neuron-specific enolase (NSE) immunosensor based on CoFe_2_O_4_@Ag nanocomposite and AuNPs@MoS_2_/rGO. Anal. Chim. Acta 1200, 339609. 10.1016/j.aca.2022.339609 35256133

[B21] Khatri R. Puri N. K. (2022a). Electrochemical biosensor utilizing dual-mode output for detection of lung cancer biomarker based on reduced graphene oxide-modified reduced-molybdenum disulfide multi-layered nanosheets. J. Mater. Res. 37, 1451–1463. 10.1557/s43578-022-00546-w

[B22] Khatri R. Puri N. K. (2022b). Electrochemical studies of biofunctionalized MoS2 matrix for highly stable immobilization of antibodies and detection of lung cancer protein biomarker. New J. Chem. 46, 7477–7489. 10.1039/d2nj00540a

[B23] Kuntamung K. Sangthong P. Jakmunee J. Ounnunkad K. (2021). A label-free immunosensor for the detection of a new lung cancer biomarker, GM2 activator protein, using a phosphomolybdic acid/polyethyleneimine coated gold nanoparticle composite. Analyst 146, 2203–2211. 10.1039/d0an02149k 33595007

[B24] Li M. Fang J. Wang C. Zhang J. Liu L. Li Y. (2022). CePO_4_/CeO_2_ heterostructure and enzymatic action of D-Fe_2_O_3_ co-amplify luminol-based electrochemiluminescence immunosensor for NSE detection. Biosens. Bioelectron. 214, 114516. 10.1016/j.bios.2022.114516 35803148

[B25] Li W. Li M. Huang Q. He X. Shen C. Hou X. (2023). Advancement of regulating cellular signaling pathways in NSCLC target therapy via nanodrug. Front. Chem. 11, 1251986. 10.3389/fchem.2023.1251986 37744063 PMC10512551

[B26] Li W. Yang Y. Ma C. Song Y. Qiao X. Hong C. (2020). A sandwich-type electrochemical immunosensor for ultrasensitive detection of multiple tumor markers using an electrical signal difference strategy. Talanta 219, 121322. 10.1016/j.talanta.2020.121322 32887059

[B27] Liu J. Liu J. Shang Y. Xu J. Wang X. Zheng J. (2022). An electrochemical immunosensor for simultaneous detection of two lung cancer markers based on electroactive probes. J. Electroanal. Chem. 919, 116559. 10.1016/j.jelechem.2022.116559

[B28] Liu Y. Si S. Dong S. Ji B. Li H. Liu S. (2021). Ultrasensitive electrochemical immunosensor for ProGRP detection based on 3D-rGO@Au nanocomposite. Microchem. J. 170, 106644. 10.1016/j.microc.2021.106644

[B29] Lu J. Hao L. Yang F. Liu Y. Yang H. Yan S. (2021). Ultrasensitive electrochemical detection of CYFRA 21-1 via *in-situ* initiated ROP signal ampli fi cation strategy. Anal. Chim. Acta 1180, 338889. 10.1016/j.aca.2021.338889 34538315

[B30] Lu Y. Futtner C. Rock J. R. Xu X. Whitworth W. Hogan B. L. M. (2010). Evidence that SOX2 overexpression is oncogenic in the lung. PLoS ONE 5, e11022. 10.1371/journal.pone.0011022 20548776 PMC2883553

[B31] Meng X. Chen X. Wu W. Zheng W. Deng H. Xu L. (2019). Electrochemiluminescent immunoassay for the lung cancer biomarker CYFRA21-1 using MoO x quantum dots. Microchim. Acta 186, 855–858. 10.1007/s00604-019-3917-4 31784817

[B32] Ning X. Xiong Q. Zhang F. He P. (2018). Simultaneous detection of tumor markers in lung cancer using scanning electrochemical microscopy. J. Electroanal. Chem. 812, 101–106. 10.1016/j.jelechem.2018.01.061

[B33] Okamura K. Takayama K. Izumi M. Harada T. Furuyama K. Nakanishi Y. (2013). Diagnostic value of CEA and CYFRA 21-1 tumor markers in primary lung cancer. Lung Cancer 80, 45–49. 10.1016/j.lungcan.2013.01.002 23352032

[B34] Page M. J. McKenzie J. E. Bossuyt P. M. Boutron I. Hoffmann T. C. Mulrow C. D. (2021). The PRISMA 2020 statement: an updated guideline for reporting systematic reviews. BMJ 372, n71. 10.1136/bmj.n71 33782057 PMC8005924

[B35] Paimard G. Shahlaei M. Moradipour P. Akbari H. Jafari M. Arkan E. (2020). An Impedimetric Immunosensor modified with electrospun core-shell nanofibers for determination of the carcinoma embryonic antigen. Sensors Actuators, B Chem. 311, 127928. 10.1016/j.snb.2020.127928

[B36] Ravi R. Zeyaullah Md. Ghosh S. Warsi M. K. Baweja R. AlShahrani A. M. (2022). Use of gold nanoparticle-silibinin conjugates: a novel approach against lung cancer cells. Front. Chem. 10, 1018759. 10.3389/fchem.2022.1018759 36311430 PMC9606463

[B37] Regiart M. Gimenez A. M. Lopes A. T. Carreño M. N. P. Bertotti M. (2020). Ultrasensitive microfluidic electrochemical immunosensor based on electrodeposited nanoporous gold for SOX-2 determination. Anal. Chim. Acta 1127, 122–130. 10.1016/j.aca.2020.06.037 32800115

[B38] Rejeeth C. Sharma A. Kannan S. Kumar R. S. Almansour A. I. Arumugam N. (2022). Label-free electrochemical detection of the cancer biomarker platelet-derived growth factor receptor in human serum and cancer cells. ACS Biomaterials Sci. Eng. 8, 826–833. 10.1021/acsbiomaterials.1c01135 34874151

[B39] Roberts A. Prakash P. Gandhi S. (2019). Graphene nanosheets as an electric mediator for ultrafast sensing of urokinase plasminogen activator receptor-A biomarker of cancer. Biosens. Bioelectron. 141, 111398. 10.1016/j.bios.2019.111398 31176112

[B40] Saltos A. Khalil F. Smith M. Li J. Schell M. Antonia S. J. (2018). Clinical associations of mucin 1 in human lung cancer and precancerous lesions. Oncotarget 9, 35666–35675. 10.18632/oncotarget.26278 30479696 PMC6235019

[B41] Saputra H. Chung J. Yoon S. Seo K. Park D. Shim Y. (2022). Disposable amperometric immunosensor with a dual monomers-based bioconjugate for granzyme B detection in blood and cancer progress monitoring of patients. Biosensensors Bioelectron. 198, 113846. 10.1016/J.BIOS.2021.113846 34871833

[B42] Satoh H. Ishikawa H. Kurishima K. Yamashita Y. T. Ohtsuka M. Sekizawa K. (2002). Cut-off levels of NSE to differentiate SCLC from NSCLC. Oncol. Rep. 9, 581–583. 10.3892/or.9.3.581 11956631

[B43] Schabath M. B. Cote M. L. (2019). Cancer progress and priorities: lung cancer. Cancer Epidemiol. Biomarkers Prev. 28, 1563–1579. 10.1158/1055-9965.EPI-19-0221 31575553 PMC6777859

[B44] Seijo L. M. Peled N. Ajona D. Boeri M. Field J. K. Sozzi G. (2019). Biomarkers in lung cancer screening: achievements, promises, and challenges. J. Thorac. Oncol. 14, 343–357. 10.1016/j.jtho.2018.11.023 30529598 PMC6494979

[B45] Sha L. Bo B. Yang F. Li J. Cao Y. Zhao J. (2022). Programmable DNA-fueled electrochemical analysis of lung cancer exosomes. Anal. Chem. 94, 8748–8755. 10.1021/acs.analchem.2c01318 35649159

[B46] Sumithra B. Jayanthi V. S. P. K. S. A. Manne H. C. Gunda R. Saxena U. Das A. B. (2020). Antibody-based biosensor to detect oncogenic splicing factor Sam68 for the diagnosis of lung cancer. Biotechnol. Lett. 42, 2501–2509. 10.1007/s10529-020-02951-9 32648188

[B47] Taheri N. Khoshsafar H. Ghanei M. Ghazvini A. Bagheri H. (2022). Dual-template rectangular nanotube molecularly imprinted polypyrrole for label-free impedimetric sensing of AFP and CEA as lung cancer biomarkers. Talanta 239, 123146. 10.1016/j.talanta.2021.123146 34942484

[B48] Tang Z. Ma Z. (2017). Multiple functional strategies for amplifying sensitivity of amperometric immunoassay for tumor markers: a review. Biosens. Bioelectron. 98, 100–112. 10.1016/j.bios.2017.06.041 28662470

[B49] Tanoue L. T. Tanner N. T. Gould M. K. Silvestri G. A. (2015). Lung cancer screening. Am. J. Respir. Crit. Care Med. 191, 19–33. 10.1164/rccm.201410-1777CI 25369325

[B50] Triphuridet N. Vidhyarkorn S. Worakitsitisatorn A. Sricharunrat T. Teerayathanakul N. Auewarakul C. (2018). Screening values of carcinoembryonic antigen and cytokeratin 19 fragment for lung cancer in combination with low-dose computed tomography in high-risk populations: initial and 2-year screening outcomes. Lung Cancer 122, 243–248. 10.1016/j.lungcan.2018.05.012 30032839

[B51] Wang M. Li J. Chen J. Zhang Y. Jia Y. Yang H. (2021). Ultrasensitive electrochemical immunosensor via RAFT polymerization signal ampli fi cation for the detection of lung cancer biomarker. J. Electroanal. Chem. 882, 114971. 10.1016/j.jelechem.2020.114971

[B52] Wang M. Lin Y. Wu S. Deng Y. Zhang Y. Yang J. (2022a). An electrochemical biosensor for PD-L1 positive exosomes based on ultra-thin two-dimensional covalent organic framework nanosheets coupled with CRISPR-Cas12a mediated signal amplification. Sensors Actuators B Chem. 362, 131813. 10.1016/j.snb.2022.131813

[B53] Wang X. Liao X. Zhang B. Chen S. Zhang M. Mei L. (2022b). Fabrication of a novel electrochemical immunosensor for the sensitive detection of carcinoembryonic antigen using a double signal attenuation strategy. Anal. Chim. Acta 1232, 340455. 10.1016/j.aca.2022.340455 36257740

[B54] Wang Y. Luo J. Liu J. Sun S. Xiong Y. Ma Y. (2019). Label-free microfluidic paper-based electrochemical aptasensor for ultrasensitive and simultaneous multiplexed detection of cancer biomarkers. Biosens. Bioelectron. 136, 84–90. 10.1016/j.bios.2019.04.032 31039491

[B55] Wang Y. Sun S. Luo J. Xiong Y. Ming T. Liu J. (2020). Low sample volume origami-paper-based graphene-modified aptasensors for label-free electrochemical detection of cancer biomarker-EGFR. Microsystems Nanoeng. 6, 32–39. 10.1038/s41378-020-0146-2 PMC8433370 34567646

[B56] Wei Z. Zhang J. Zhang A. Wang Y. Cai X. (2017). Electrochemical detecting lung cancer-associated antigen based on graphene-gold nanocomposite. Molecules 22, 392–399. 10.3390/molecules22030392 28257099 PMC6155348

[B57] Xie F. T. Li Y. L. Guan Y. Liu J. W. Yang T. Mao G. J. (2022). Ultrasensitive dual-signal electrochemical ratiometric aptasensor based on Co-MOFs with intrinsic self-calibration property for Mucin 1. Anal. Chim. Acta 1225, 340219. 10.1016/j.aca.2022.340219 36038234

[B58] Yadav N. Dahiya T. Chhillar A. Rana J. Saini H. (2022). Nanotechnology in cancer diagnostics and therapeutics: a review. Curr. Pharm. Biotechnol. 23, 1556–1568. 10.2174/1389201023666211222165508 34951360

[B59] Yang H. Bao J. Huo D. Zeng Y. Wang X. Samalo M. (2021). Au doped poly-thionine and poly-m-Cresol purple: synthesis and their application in simultaneously electrochemical detection of two lung cancer markers CEA and CYFRA21-1. Talanta 224, 121816. 10.1016/j.talanta.2020.121816 33379041

[B60] Yi R. Li Y. Wang S. Liu Q. Dong H. Liu S. (2022). A neuron-specific enolase electrochemical immunosensor based on rGO/Cu 8 Ni 2 nanocomposite with enhanced catalytic activity. J. Electrochem. Soc. 169, 067509. 10.1149/1945-7111/ac7a61

[B61] Yola M. L. Atar N. Özcan N. (2021). A novel electrochemical lung cancer biomarker cytokeratin 19 fragment antigen 21-1 immunosensor based on Si3N4/MoS2incorporated MWCNTs and core-shell type magnetic nanoparticles. Nanoscale 13, 4660–4669. 10.1039/d1nr00244a 33620353

[B62] You Q. Zhuang L. Chang Z. Ge M. Mei Q. Yang L. (2022). Hierarchical Au nanoarrays functionalized 2D Ti2CTx MXene membranes for the detection of exosomes isolated from human lung carcinoma cells. Biosens. Bioelectron. 216, 114647. 10.1016/j.bios.2022.114647 36029661

[B63] Zeng Y. Bao J. Zhao Y. Huo D. Chen M. Qi Y. (2018). A sandwich-type electrochemical immunoassay for ultrasensitive detection of non-small cell lung cancer biomarker CYFRA21-1. Bioelectrochemistry 120, 183–189. 10.1016/j.bioelechem.2017.11.003 29289826

[B64] Zhang J. Shen Q. Zhou Y. (2021a). Quantification of tumor protein biomarkers from lung patient serum using nanoimpact electrochemistry. ACS Sensors 6, 2320–2329. 10.1021/acssensors.1c00361 34033456

[B65] Zhang Q. Li X. Qian C. Dou L. Cui F. Chen X. (2018). Label-free electrochemical immunoassay for neuron specific enolase based on 3D macroporous reduced graphene oxide/polyaniline film. Anal. Biochem. 540–541, 1–8. 10.1016/j.ab.2017.10.009 29113785

[B66] Zhang R. Rejeeth C. Xu W. Zhu C. Liu X. Wan J. (2019). Label-free electrochemical sensor for CD44 by ligand-protein interaction. Anal. Chem. 91, 7078–7085. 10.1021/acs.analchem.8b05966 30942566

[B67] Zhang R. Zhang J. Tan F. Yang D. Wang B. Dai J. (2022a). Multi-channel AgNWs-doped interdigitated organic electrochemical transistors enable sputum-based device towards noninvasive and portable diagnosis of lung cancer. Mater. Today Bio 16, 100385. 10.1016/j.mtbio.2022.100385 PMC9386496 35991625

[B68] Zhang W. Tian Z. Yang S. Rich J. Zhao S. Klingeborn M. (2021b). Electrochemical micro-aptasensors for exosome detection based on hybridization chain reaction amplification. Microsystems Nanoeng. 7, 63–68. 10.1038/s41378-021-00293-8 PMC8433316 34567775

[B69] Zhang Y. Zhu H. Ying Z. Gao X. Chen W. Zhan Y. (2022b). Design and application of metal organic framework ZIF-90-ZnO-MoS2Nanohybrid for an integrated electrochemical liquid biopsy. Nano Lett. 22, 6833–6840. 10.1021/acs.nanolett.2c01613 35819288

[B70] Zhao H. Liu T. Cui L. Li Y. Yang F. Zhang X. (2021). Label-free and dual-amplified electrochemical bioanalysis of MUC1 based on an inorganic-organic polymer hybrid mimic peroxidase (AuNPs@Cu7S4@Cu/Mn-AzoPPOP) and catalytic hairpin assembly. Sensors Actuators, B Chem. 345, 130332. 10.1016/j.snb.2021.130332

[B71] Zhou Y. Li M. Wang H. Sun S. X. (2022). Dual-signal amplified electrochemical biosensor based on eATRP and PEI for early detection of lung cancer. Bioelectrochemistry 148, 108224. 10.1016/j.bioelechem.2022.108224 36029762

[B72] Zhu C. Yang G. Li H. Du D. Lin Y. (2015). Electrochemical sensors and biosensors based on nanomaterials and nanostructures. Anal. Chem. 87, 230–249. 10.1021/ac5039863 25354297 PMC4287168

